# Synthesis of dye/fluorescent functionalized dendrons based on cyclotriphosphazene

**DOI:** 10.3762/bjoc.7.186

**Published:** 2011-11-28

**Authors:** Aurélien Hameau, Sabine Fuchs, Régis Laurent, Jean-Pierre Majoral, Anne-Marie Caminade

**Affiliations:** 1 CNRS, LCC (Laboratoire de Chimie de Coordination), 205, route de Narbonne, BP 44099, F-31077 Toulouse, France; 2 Université de Toulouse; UPS, INPT, LCC, F-31077 Toulouse, France

**Keywords:** dabsyl, dansyl, dendrimer, dendron, fluorescence

## Abstract

Functionalized phenols based on tyramine were synthesized in order to be selectively grafted on to hexachlorocyclotriphosphazene, affording a variety of functionalized dendrons of type AB_5_. The B functions comprised fluorescent groups (dansyl) or dyes (dabsyl), whereas the A function was provided by either an aldehyde or an amine. The characterization of these dendrons is reported. An unexpected behaviour of a fluorescent and water-soluble dendron based on dansyl groups in mixtures of dioxane/water was observed.

## Introduction

Dendrimers constitute an important group of hyperbranched macromolecules, pertaining both to the field of molecular chemistry thanks to their perfectly defined structure due to their step-by-step synthesis, and to the field of polymers due to their repetitive structure. The numerous terminal groups of dendrimers are responsible for most of their properties for instance in catalysis, for the elaboration of materials, or in biology [1–2]. Dendrons are also branched macromolecules, reminiscent of dendrimers, but which possess one functional group at the level of the core, in addition to the terminal groups [3]. Several hundreds of publications have reported the synthesis, the study of photophysical properties and several uses of fluorescent dendrimers, emphasizing in particular their role as light-harvesters [4] for organic light-emitting diodes (OLEDs) [5] and as tools in biology [6]. Many different types of fluorophores have been grafted as terminal groups on to dendrimers. In particular the dansyl group has been frequently used to functionalize poly(propyleneimine) [7–8], poly(lysine) [9], poly(amidoamine) [10], and poly(melamine) [11] dendrimers. It has been shown that no interaction occurs between these terminal fluorophores, at least up to generation 5, whereas protonation induces drastic changes in the absorption and emission bands [12]. The dansyl group has frequently been employed as the core of dendrons, but very rarely as a terminal group. Meanwhile, functionalized dendrons (dendrimeric wedges) are potentially interesting for elaboration of the original dendrimeric architectures [13–14], or for labelling materials or biological entities. In many cases, it is desirable to concentrate a maximum number of functional groups in a small compound (a small size is particularly important so as not to disturb biological systems); this can be attained if the core of the dendron possesses several functions.

We report here the use of hexachlorocyclotriphosphazene (N_3_P_3_Cl_6_) for such a purpose, which allows the gathering of five fluorescent dansyl groups in a small dendron, thanks to the specific functionalization of N_3_P_3_Cl_6_ [15]. The method elaborated is also useful for the grafting of dyes, as will be illustrated with the dabsyl dye. We also paid particular attention to the nature of the remaining function (usable for the grafting of the dendron). We choose an aldehyde and a primary amine, both being well-known for their broad and versatile reactivities.

## Results and Discussion

### Synthesis of functionalized dendrons

**Scheme 1:**
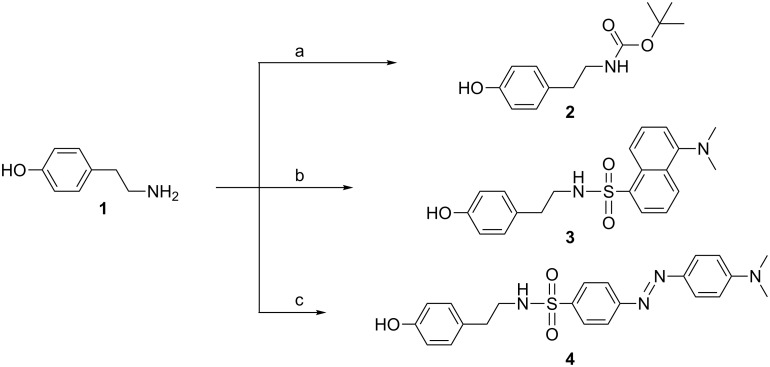
Synthesis of functionalized phenols. Reagents and conditions: a) Boc_2_O, 1.0 equiv, THF, 0 °C → rt, 4 h. b) Dansyl chloride, 1.1 equiv, MeOH, rt, 6 h. c) Dabsyl chloride, 1.0 equiv, CH_2_Cl_2_ and MeOH, rt, 16 h.

The first step of the strategy entails the non-symmetrical functionalization of hexachlorocyclotriphosphazene (N_3_P_3_Cl_6_) in order to synthesize AB_5_-type compounds, where A is the function usable, for instance, for the coupling with another dendron, and the B functions are either the dyes or the fluorescent groups. Due to the known, relatively easy functionalization of N_3_P_3_Cl_6_ by phenols, and the stability of the resulting compounds, we chose functionalized phenols as the A and B functions. Functionalized phenols **2**, **3** and **4** are synthesized from tyramine (**1**). The *N*-Boc protected tyramine **2** is synthesized by using Boc_2_O. Tyramine cannot be used directly to introduce the amine functionality, because both the phenol and the amine group of tyramine are able to react with N_3_P_3_Cl_6_, thus the NH_2_ group has to be protected. The fluorescent *N*-dansyl tyramine (**3**) is obtained by reaction of tyramine (**1**) with dansyl chloride. The dye, dabsylated tyramine **4** is analogously obtained from dabsyl chloride (Scheme 1).

**Scheme 2:**
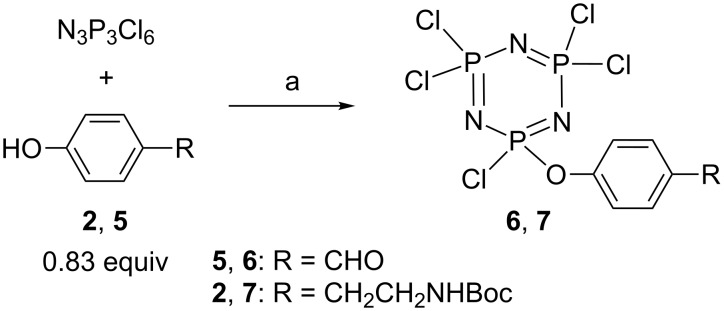
Single substitution of hexachlorocyclotriphosphazene. Reagents and conditions: a) Cs_2_CO_3_, 1.67 equiv, THF, rt, 16 h.

Having in hand four functionalized phenols (the fourth is 4-hydroxybenzaldehyde, (**5**)), the synthesis of AB_5_-type compounds starting from N_3_P_3_Cl_6_ could be accomplished either by reacting first one A then five B functions, or by reacting first five B, then one A function. The products from attempts made with phenol **5** were found to be easier to purify when the first method was used. A slight substoichiometric amount of phenol was used, in order to avoid disubstitution with phenols **5** or **2** (the A function). The monosubstituted products **6** and **7** were isolated in moderate yield after work-up (51.7 and 48.3%, respectively) (Scheme 2). Both compounds were characterized by multinuclear NMR, in particular by ^31^P NMR, which displayed in both cases the presence of one triplet and one doublet (^2^*J* (P,P) = 60 Hz), showing the unsymmetrical nature of these compounds.

**Scheme 3:**
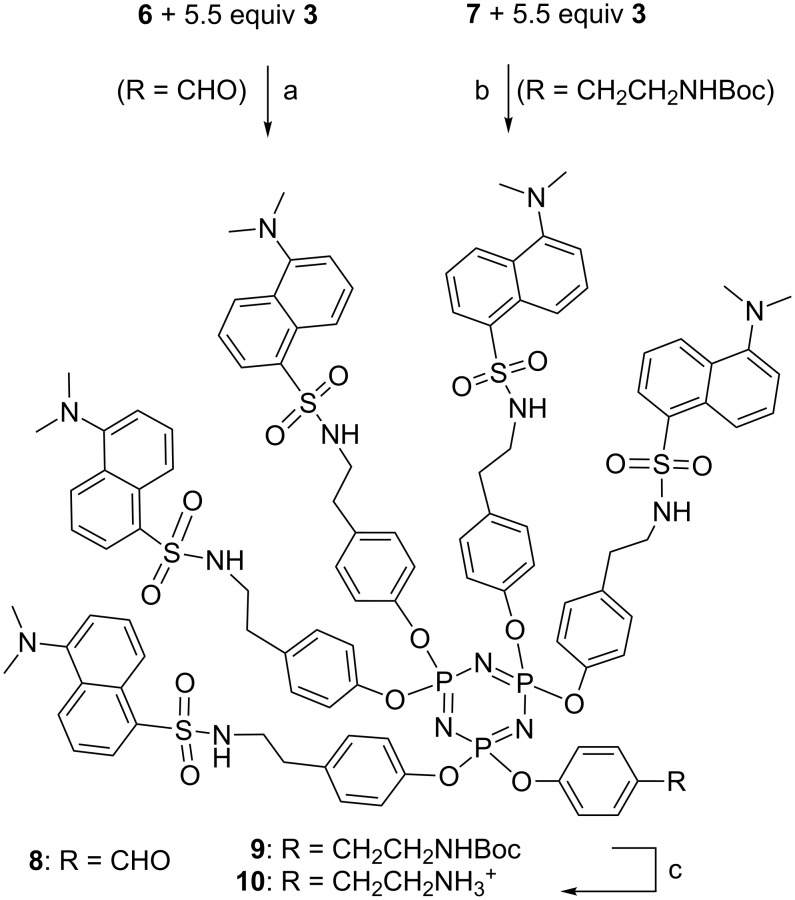
Synthesis of dansylated dendrons. Reagents and conditions: a) Cs_2_CO_3_, 11 equiv, THF, rt, 48 h. b) Cs_2_CO_3_, 11 equiv, THF, rt, 48 h. c) TFA (25% v:v), CH_2_Cl_2_, rt, 1 h, evaporation, repeated once.

The next step involves the grafting of five equivalents of the dansyl phenol **3** on compounds **6** or **7**, to afford dendrons **8** or **9**, respectively. These were isolated in good yields after work-up (69.2 and 56.6%), and characterized by mass spectrometry and multinuclear NMR. The ^31^P NMR spectra generally consisted of multiplets, due to the non-symmetrical nature of these compounds. The aldehyde group is directly usable for grafting, as we have already demonstrated for the elaboration of Janus dendrimers [12], but the Boc protection of compound **9** must be removed in order to afford the amine group. The Boc protection of tyramine was cleaved by using trifluoroacetic acid. The reaction was monitored by ^1^H NMR, which showed the total disappearance of the signal corresponding to Boc (δ = 1.41 ppm) after the repetition of the cleavage step. The dendron **10** was isolated as a salt in 98.1% yield after work-up (Scheme 3).

**Scheme 4:**
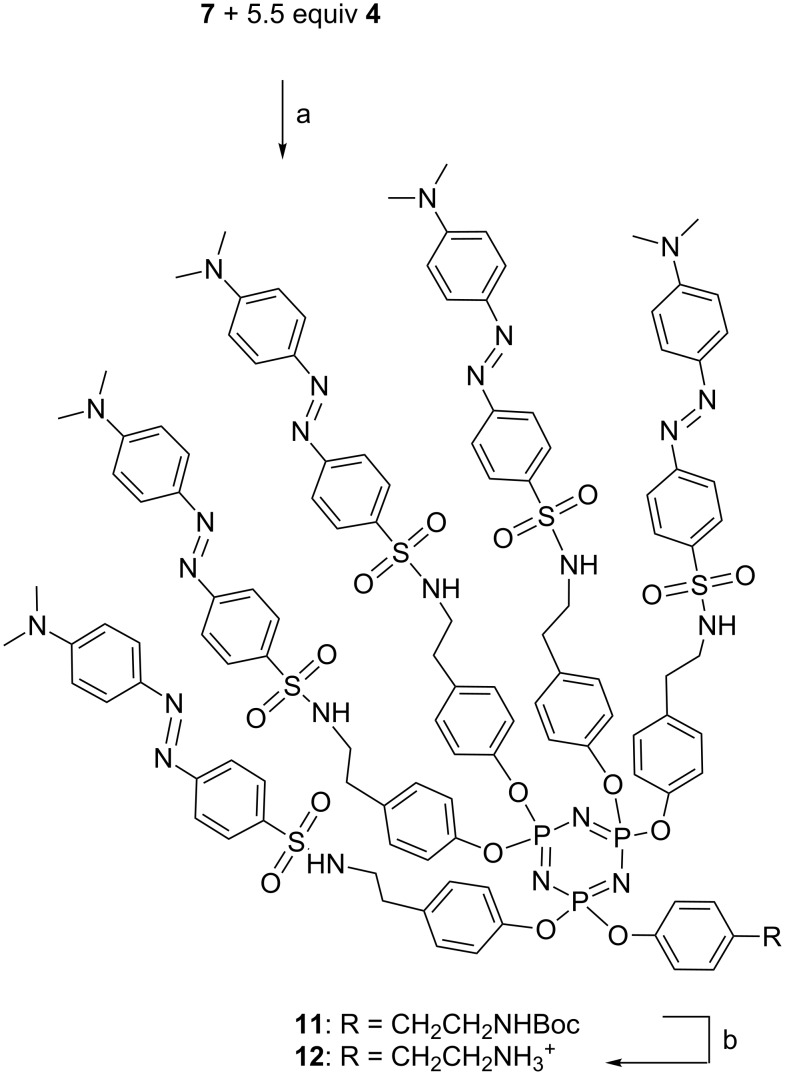
Synthesis of dabsylated dendrons. Reagents and conditions: a) Cs_2_CO_3_, 11 equiv, THF, rt, 48 h. b) TFA (10% v:v), CH_2_Cl_2_, rt, 1 h, evaporation, repeated once.

The same procedure was applied to afford the dabsylated dendron **11** in 43.3% yield from the dabsyl phenol **4** and the Boc-protected cyclotriphosphazene derivative **7**. The deprotection was carried out as described above to afford the dendron **12** (Scheme 4).

### UV–vis absorption spectra and fluorescence properties

**Figure 1:**
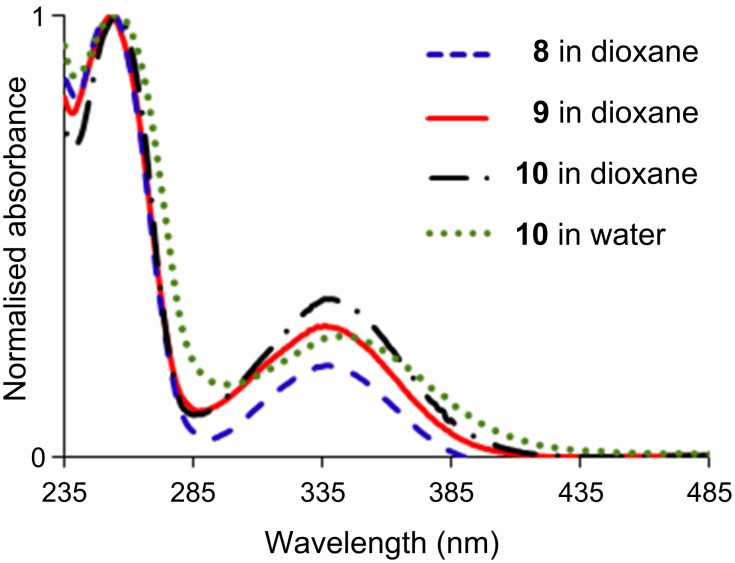
UV–vis spectra of compounds **8**–**10**.

**Figure 2:**
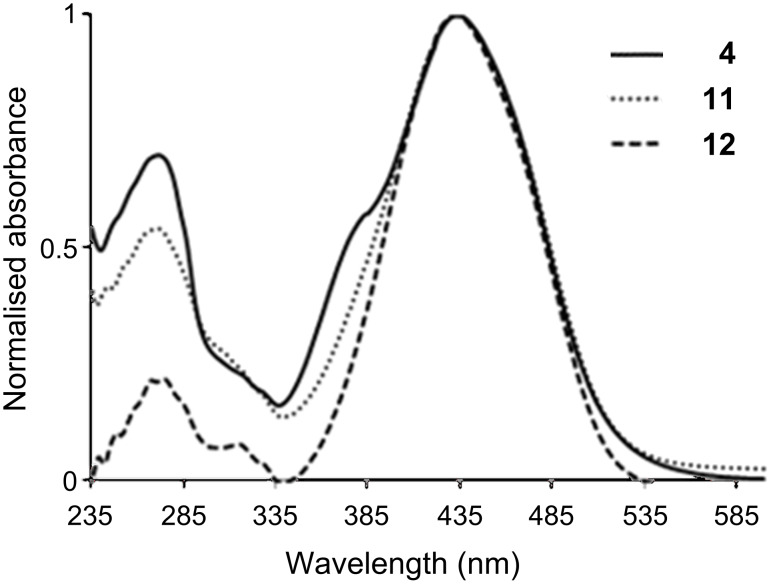
UV–vis spectra of compounds **4**, **11** and **12** in dioxane.

Having in hand five different dendrons, we recorded the UV–vis spectra in 1,4-dioxane. As expected, each family affords a coherent series of spectra. In the case of the dansyl derivatives (**8**–**10**), two absorption peaks are observed at 253 and ca. 338 nm (Table 1). Compound **10** is also soluble in water; its UV–vis spectrum in this solvent is analogous to that in dioxane. It is important to note that there is no peak at 280 nm [12], meaning that no protonation of dansyl occurred, even in the case of derivative **10** in water (Figure 1). In the case of the dabsyl derivatives, there is a large signal at 434 nm, the high intensity of which corresponds to its dye properties, and a smaller one at 271 nm. The wavelength of the signal in the visible part remains constant, indicating here also that there is no protonation of dabsyl, even for dendron **12** (Figure 2) [16].

**Table 1:** UV–vis characteristics and fluorescence characteristics of dendrons **8**–**10** in 1,4-dioxane.

Dendron	λ_max (1)_ (nm)	ε _(1)_ (L·mol^−1^·cm^−1^)	λ_max (2)_ (nm)	ε _(2)_ (L·mol^−1^·cm^−1^)	λ_em,max_^a^ (nm)	Quantum yield Φ (%)

**8**	253	103 × 10^3^	338	21 × 10^3^	493	47
**9**	253	71 × 10^3^	336	21 × 10^3^	491	55
**10**	255	69 × 10^3^	336	21 × 10^3^	492	51

^a^Excitation wavelength 347 nm.

**Figure 3:**
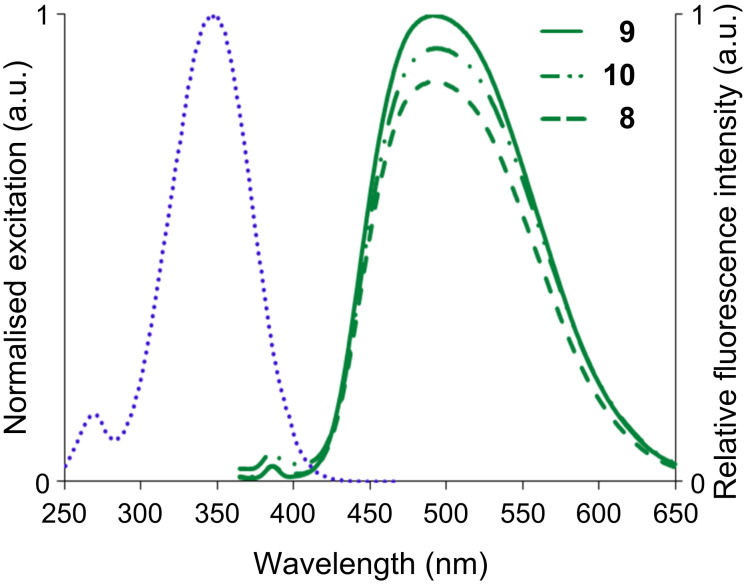
Excitation (left) and emission (right) spectra of compounds **8**–**10** in 1,4-dioxane (the excitation spectra of the 3 compounds are entirely superimposed).

All the dansyl dendrons (**8**–**10**) are fluorescent; their excitation and fluorescence spectra were recorded in dioxane. The excitation and emission spectra are given in Figure 3. It appears that the fluorescence quantum yield is slightly sensitive to the nature of the A functionality (Table 1).

**Figure 4:**
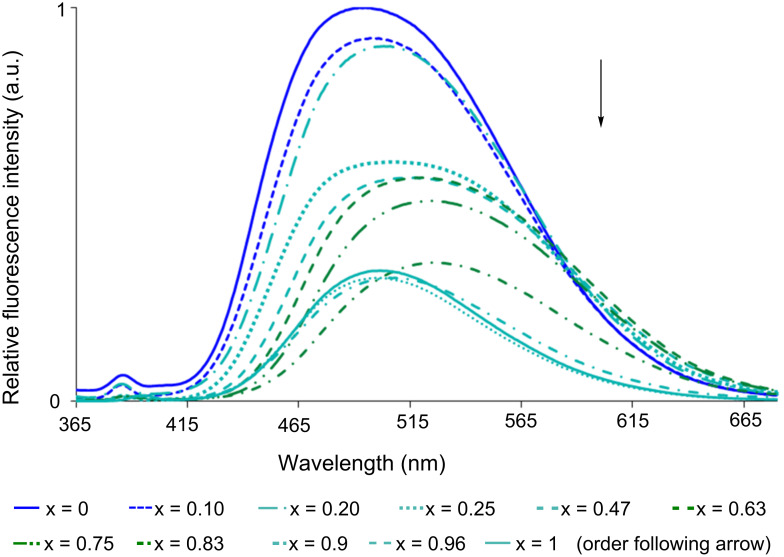
Emission spectra (λ_exc_ = 347 nm) of compound **10** in mixtures water/dioxane with different molar fractions of water (x) in 1,4-dioxane.

Dendrons **8** and **9** are soluble in organic solvents, as is dendron **10**, but in the case of **10** it is also soluble in water in contrast to the others, thus we also studied its fluorescence in water. We observed a notable decrease of its fluorescence quantum yield (Φ) in water upon going from pure dioxane (Φ = 51) to pure water (Φ = 17), which cannot be related to protonation, as indicated from the UV–vis spectrum [10]. An analogous phenomenon was already described for a dansyl derivative of adenosine in solution in water or in pyridine, and was ascribed to the solvent polarity, which modifies the interactions between both parts of the molecule [17]. In our case, we may consider that the structure of dendron **10** in solution in dioxane is more expanded than in water, which should bring the hydrophobic moieties (the dansyl groups) closer, favoring the quenching. A slight red-shift of the maxima of emission (from 492 to 501 nm) was also observed (Table 2 and solid lines in Figure 4), which might be related to changes in the polarity of the solvent [18]. Given that dioxane and water are miscible, we decided to measure the fluorescence spectra of compound **10** in mixtures of both solvents in various proportions. Even a small quantity of water in dioxane (molar fraction 0.1) is sufficient to induce a large drop in the fluorescence intensity. To our surprise, increasing the quantity of water induces an increased red-shift of the fluorescence wavelength up to a molar fraction of 0.8 in water (from 492 to 524 nm), then a dramatic blue-shift is observed as the quantity was increased up to pure water (501 nm) (Figure 4).

**Table 2:** Viscosity (η) [17], maximum of absorption (λ_max_), fluorescence emission (excitation wavelength 347 nm) (λ_em_), and quantum yield (Φ) (reference: quinine sulfate) of compound **10** in mixtures of water/dioxane with different molar fractions (x) of water in 1,4-dioxane.

x_water_	water/dioxane (% v/v)	η (mPa·s)	λ_max (1)_ (nm)	λ_max (2)_ (nm)	λ_em,max_ (nm)	quantum yield, Φ (%)

0	0/100	1.31	255	336	492	51
0.10	2/98	1.35	248	336	499	47
0.20	5/95	1.45	250	336	505	46
0.25	7/93	1.53	252	338	507	31
0.34	10/90	1.66	250	339	510	28
0.47	15/85	1.86	254	339	514	29
0.54	20/80	1.98	249	337	516	29
0.63	26/74	2.2	252	338	520	29
0.75	39/61	2.3	253	338	524	26
0.83	50/50	2.2	250	336	524	18
0.87	59/41	2.01	249	339	521	18
0.89	62/38	1.92	251	338	514	18
0.9	65/35	1.86	253	341	506	16
0.94	77/23	1.50	252	339	501	17
0.96	83/17	1.31	253	341	501	16
1	100/0	1.01	256	340	501	17

**Figure 5:**
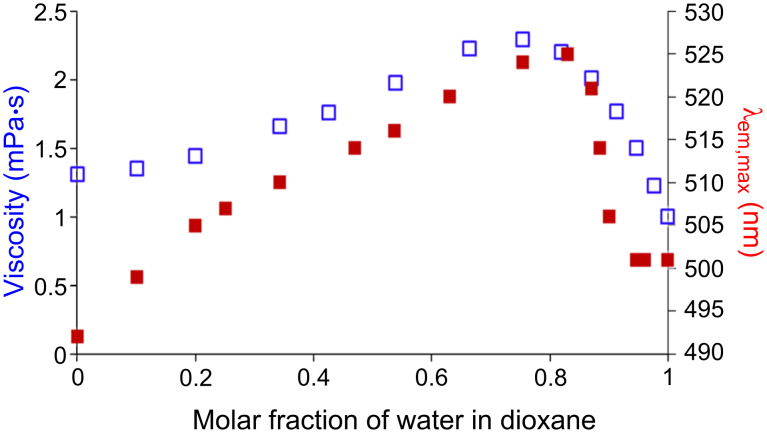
λ_em,max_ of **10** in mixture water/dioxane vs molar fraction of water in dioxane (filled squares) and viscosity dependence [19] of a mixture water/dioxane vs molar fraction of water in dioxane (empty squares).

This unexpected behavior must be related to the variation of the viscosity of the mixtures dioxane/water, which is maximal at the molar fraction 0.8 [19]. Figure 5 displays the comparison between the maxima wavelength of emission of dendron **10** with the viscosity of the mixture dioxane/water. A good correlation between both curves is observed, showing that the viscosity is indeed the most important criterion that needs to be taken into account to explain the phenomenon observed.

## Conclusion

We have synthesized several new functionalized dendrons based on cyclotriphosphazene, bearing both one functional group and several dyes (dabsyl) or fluorescent groups (dansyl). In particular, new brightly fluorescent dendrons were synthesized and their fluorescence properties studied. One of them, **10** is both soluble in organic solvents and in water, and displays novel behaviour in mixtures of solvent (dioxane/water). Variations in the fluorescence quantum yield of its dansyl groups must be related to differences in the polarity of the solvents, enhanced by the hydrophobic nature of the dansyl part, and to their close proximity in the dendron. Variations of the emission wavelengths must be related to the variations in the viscosity of the mixtures. Such findings demonstrate that dendron **10** is a new sensing element that is particularly sensitive in water solutions, and which may find uses in the fields of materials or biology.

## Supporting Information

File 1 Experimental details.

File 2 Spectral details.

## References

[1] Caminade A-M, Turrin C-O, Laurent R (2011). Dendrimers: Towards Catalytic, Material and Biomedical Uses.

[2] Astruc D, Boisselier E, Ornelas C (2010). Chem Rev.

[3] Grayson S M, Fréchet J M J (2001). Chem Rev.

[4] Adronov A, Fréchet J M J (2000). Chem Commun.

[5] Hwang S-H, Moorefield C N, Newkome G R (2008). Chem Soc Rev.

[6] Caminade A-M, Hameau A, Majoral J-P (2009). Chem–Eur J.

[7] Vögtle F, Gestermann S, Kauffmann C, Ceroni P, Vicinelli V, Balzani V (2000). J Am Chem Soc.

[8] Hahn U, Gorka M, Vögtle F, Vicinelli V, Ceroni P, Maestri M, Balzani V (2002). Angew Chem, Int Ed.

[9] Vicinelli V, Ceroni P, Maestri M, Balzani V, Gorka M, Vögtle F (2002). J Am Chem Soc.

[10] Wang B-B, Zhang X, Jia X-R, Li Z-C, Ji Y, Yang L, Wei Y (2004). J Am Chem Soc.

[11] Zhang W, Tichy S E, Pérez L M, Maria G C, Lindahl P A, Simanek E E (2003). J Am Chem Soc.

[12] Vögtle F, Gestermann S, Kauffmann C, Ceroni P, Vicinelli V, De Cola L, Balzani V (1999). J Am Chem Soc.

[13] Hawker C J, Fréchet J M J (1990). J Am Chem Soc.

[14] Fuchs S, Pla-Quintana A, Mazères S, Caminade A-M, Majoral J-P (2008). Org Lett.

[15] Maraval V, Caminade A-M, Majoral J-P, Blais J-C (2003). Angew Chem, Int Ed.

[16] Siiman O, Lepp A (1984). J Phys Chem.

[17] Skorka G, Shuker P, Gill D, Zabicky J, Parola A H (1981). Biochemistry.

[18] Gao L, Song Q, Huang X, Huang J (2008). J Colloid Interface Sci.

[19] Omota L-M, Iulian O, Ciocîrlan O, Niţă I (2008). Rev Roum Chim.

