# Supplementary material for: Synthesis of dye/fluorescent functionalized dendrons based on cyclotriphosphazene
Source: Beilstein J Org Chem. 2011 Nov 28;7:1577–83. doi: 10.3762/bjoc.7.186 (PMC3252861; doi:10.3762/bjoc.7.186)
Supplement: File 1 — Experimental details. [file Beilstein_J_Org_Chem-07-1577-s001.pdf]

Supporting Information

for

Synthesis of dye/fluorescent functionalized dendrons based on cyclotriphosphazene

Aurélien Hameau[1,2], Sabine Fuchs[1,2], Régis Laurent[1,2], Jean-Pierre Majoral*[1,2] and Anne-Marie Caminade*[1,2]

Address: [1]CNRS, LCC (Laboratoire de Chimie de Coordination), 205, route de Narbonne, BP 44099, F-31077 Toulouse, France and [2]Université de Toulouse; UPS, INPT, LCC, F-31077 Toulouse, France

Email: Anne-Marie Caminade - caminade@lcc-toulouse.fr; Jean-Pierre Majoral - majoral@lcc-toulouse.fr

* Corresponding author

Dedicated to the memory of our friend and former PhD student Dr Yiqian Wei who regrettably passed away on February 6th, 2011.

Experimental details

General. All manipulations were carried out with standard high-vacuum and dry-argon techniques. The solvents were freshly dried and distilled (THF and ether over sodium/benzophenone, pentane and CH_2Cl_2 over phosphorus pentoxide). ^{1}H, ^{13}C, and ^{31}P NMR spectra were recorded with Bruker ARX250, AV300, AV500 spectrometers. All spectra were measured at 25 °C in the indicated deuterated solvents. Proton and carbon chemical shifts (δ) are reported in ppm and coupling constants (*J*) are reported in Hertz (Hz). The signals in the spectra are described as s (singlet), d (doublet), t (triplet), and m (multiplet). References for NMR chemical shifts are 85% H_3PO_4 for ^{31}P NMR and $SiMe_4$ for ^{1}H and ^{13}C NMR. The assignment of ^{1}H and ^{13}C NMR signals was done by using Jmod, two-dimensional HMBC and HMQC, broadband or CW ^{31}P decoupling experiments, when necessary. Mass spectrometry (IS: ion spray) was carried out with API 365 of PE Sciex.

Chemicals were purchased from Aldrich, and were used without further purification, except for $P_3N_3Cl_6$, which was recrystallized from hexane. Organic solvents were dried and distilled by following routine procedures. Purifications by flash column chromatography were performed with silica gel (50 µm). TLCs were performed on silica gel 60 F254 plates and detection was carried out under UV light.

Synthesis of 2. 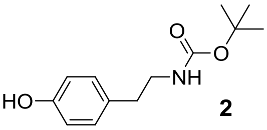

2

A suspension of tyramine (**1**) (4.12 g, 30.0 mmol) in THF was cooled to 0 °C and Boc_2O (6.54 g, 30.0 mmol) was added at once under vigorous stirring. The reaction mixture was stirred for 4 h, during which it was allowed to warm up to rt slowly. After complete reaction (TLC-monitored) the solvent was evaporated and the residue was redissolved in ethyl acetate. The organic phase was washed once with saturated $NaHCO_3$ solution and twice with brine. It was dried over $MgSO_4$, the solvent was removed in vacuo and the crude product purified by column chromatography (silica gel, hexane/ethyl acetate (4:1/v:v) as eluent). The procedure yielded the Boc-protected tyramine **2** (6.12 g, 25.8 mmol, 86.0%) as colourless crystals. R_f 0.14 (hexane/ethyl acetate (3:1/v:v)).
^{1}H NMR (250 MHz, $CDCl_3$): δ 1.45 (s, 9H, $C(CH_3)_3$); 2.68 (t, ^{3}J = 6.9 Hz, 2H, CH_2Ar), 3.33 (m, 2H, CH_2N), 4.77 (br m, 1H, NH), 6.80 (d, ^{3}J = 8.3 Hz, 2H, Ar*H*), 6.99 (d, ^{3}J = 8.3 Hz, 2H, Ar*H*), 7.54 (br s, 1H, OH); ^{13}C {^{1}H} NMR (62.9 MHz, $CDCl_3$): δ 28.44 ($C(CH_3)_3$), 35.20 (CH_2Ar), 42.10 (CH_2N), 79.83 ($C(CH_3)_3$), 115.56 (Ar*C*), 129.80 (Ar*C*), 130.00 (Ar*C*), 155.01 (Ar*C*), 156.48 (CO); EIMS *m*/*z* (%): 237 (6.8) $[M]^+$, 181 (24.8) $[C_9H_{11}NO_3]^+$, 120 (100) $[C_8H_8O]^+$.

Synthesis of 3. 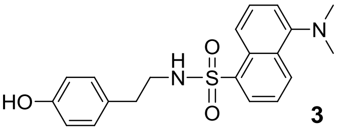 **3**

A solution of tyramine (**1**) (1.10 g, 8.00 mmol) in abs. MeOH was added dropwise to a solution of dansyl chloride (2.37 g, 8.80 mmol) in CH_2Cl_2 under vigorously stirring at rt. The highly fluorescent solution was stirred for additional 6 h at rt and during that time protected from UV light. After complete reaction (TLC-monitored), the reaction mixture was washed once with brine, once with saturated $NaHCO_3$ solution, and once again with brine. The organic phase was collected, dried over $MgSO_4$, and the solvent was evaporated in vacuo. The crude product was purified by column chromatography (silica gel, CH_2Cl_2 containing 2% MeOH as eluent) to yield the dansylated tyramine **3** (2.37 mg, 6.41 mmol, 80.1%) as a yellow-green, fluorescent solid (foam). R_f 0.47 (CH_2Cl_2/MeOH (95:5 v:v)).
^{1}H NMR (250 MHz, $CDCl_3$): δ 2.55 (t, ^3J = 6.9 Hz, 2H, CH_2Ar), 2.88 (s, 6H, $N(CH_3)_2$), 3.11 (m, 2H, CH_2N), 4.59 (t, ^3J = 6.0 Hz, 1H, NH), 5.09 (br s, 1H, OH), 6.60 (d, ^3J = 8.8 Hz, 2H, Ar*H*), 6.76 (d, ^3J = 8.3 Hz, 2H, Ar*H*), 7.17 (d, ^3J = 6.9 Hz, 1H, ArH_{Dns}), 7.49 (m, 2H, ArH_{Dns}), 8.12 (d, ^3J = 8.8 Hz, 1H, ArH_{Dns}), 8.21 (d, ^3J = 7.4 Hz, 1H, ArH_{Dns}), 8.53 (d, ^3J = 8.3 Hz, 1H, ArH_{Dns}); ^{13}C {^{1}H} NMR (62.9 MHz, $CDCl_3$): δ 34.67 (CH_2Ar), 44.48 (CH_2N), 45.45 ($N(CH_3)_2$), 115.23 (Ar*C*), 115.50 (ArC_{Dns}), 118.65 (ArC_{Dns}), 123.21 (Ar*C*), 128.45 (ArC_{Dns}), 129.23 (ArC_{Dns}), 129.45 (ArC_{Dns}), 129.63 (ArC_{Dns}), 129.71 (ArC_{Dns}), 130.31 (ArC_{Dns}), 130.46 (ArC_{Dns}), 134.39 (Ar*C*), 151.82 (ArC_{Dns}), 154.46 (Ar*C*). MS (IS, positive, MeOH) *m*/*z*: 409 $[M + K]^+$, 393 $[M + Na]^+$, 371 $[M + H]^+$; monoisotopic mass calcd for $C_{20}H_{23}N_2O_3S^+$, 371.14; found, 371.05.

Synthesis of 4. 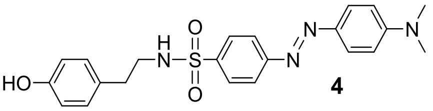 **4**

To a solution of dabsyl chloride (2.27 g, 7.00 mmol) in dry CH_2Cl_2 a solution of tyramine (**1**) (960 mg, 7.00 mmol) in dry MeOH was added dropwise at rt. The reaction mixture was stirred for an additional 16 h at rt and continuously checked by TLC. After complete reaction the solvent was removed in vacuo and the crude product redissolved in CH_2Cl_2. The resulting solution was washed once with water, once with saturated $NaHCO_3$ solution, and again three times with water. The organic phase was collected and dried over $MgSO_4$. After evaporation of the solvent the crude product was purified by column chromatography (silica gel, CH_2Cl_2 containing 1% MeOH as eluent). The procedure gave the dabsylated tyramine **4** (2.50 g, 5.89 mmol, 84.1%) as a red-orange solid. R_f 0.31 (CH_2Cl_2/MeOH (97:3 v:v)).
^{1}H NMR (250 MHz, acetone-d_6): δ 2.70 (t, ^3J = 7.5 Hz, 2H, CH_2Ar), 3.15 (m, 6H + 2H, $N(CH_3)_3$ + CH_2N), 6.73 (d, ^3J = 8.4 Hz, 2H, Ar*H*), 6.88 (d, ^3J = 9.1 Hz, 2H, ArH_{Dbs}), 7.00 (d, ^3J = 8.4 Hz, 2H, Ar*H*), 7.89 (d, ^3J = 9.1 Hz, 2H, ArH_{Dbs}), 8.23 (m, 4H, ArH_{Dbs}); ^{13}C {^{1}H} NMR (62.9 MHz, acetone-d_6): δ 35.76 (CH_2), 40.19 (CH_2), 45.70 ($N(CH_3)_3$), 112.26 (Ar*C*), 115.91 (Ar*C*), 123.03 (Ar*C*), 226.20 (Ar*C*), 128.79 (Ar*C*), 130.07 (Ar*C*), 141.56 (Ar*C*), 141.62 (Ar*C*), 144.08 (Ar*C*), 154.17 (Ar*C*), 155.90 (Ar*C*), 156.69 (Ar*C*).

MS (IS, positive, CH_3CN) *m*/*z*: 463 $[M + K]^+$, 447 $[M + Na]^+$, 425 $[M + H]^+$, 299, 224 $[M + 2Na]^{2+}$; monoisotopic mass calcd for $C_{22}H_{25}N_4O_3S^+$, 425.17; found, 425.25.

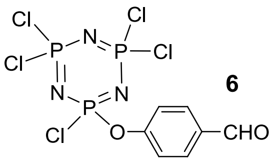

6

Synthesis of 6.

To a solution of 4-hydroxybenzaldehyde (1.22 g, 10.0 mmol) in THF, Cs_2CO_3 (6.52 g, 20 mmol) and the core $N_3P_3Cl_6$ (4.17 g, 12 mmol) were added. The reaction mixture was stirred for 16 h at rt, and the progress of the reaction was monitored by TLC. After complete reaction the salts were sedimented and the supernatant was collected. The solvent was removed in vacuo and the crude product was purified by column chromatography (silica gel, hexane/ethyl acetate (4:1 v:v) as eluent). The procedure gave the AB_5 core **6** (2.24 g, 5.17 mmol, 51.7%) as a colorless, crystalline solid. R_f 0.50 (hexane/ethyl acetate (3:1 v: v)).

^{31}P {^1H} NMR (81.0 MHz, $CDCl_3$): δ 15.18 (t, ^2J (P,P) = 62.5 Hz, PO), 26.00 (d, ^2J (P,P) = 61.0 Hz, PCl_2); ^1H NMR (250 MHz, $CDCl_3$): δ 7.44 (d, ^3J = 8.3 Hz, 2H, Ar*H*), 7.96 (d, ^3J = 8.3 Hz, 2H, Ar*H*), 10.01 (s, 1H, CHO); ^{13}C {^1H} NMR (62.9 MHz, $CDCl_3$): δ 122.15 (d, ^2J (C,P) = 5.3 Hz, Ar*C*), 131.69 (s, Ar*C*), 134.61 (s, Ar*C*), 153.44 (s, Ar*C*), 190.57 (s, CO); MS (CI, $-NH_4^+$) *m*/*z* (%): 452 (7.3) $[M + NH_4]^+$, 434 (100) $[M + H]^+$.

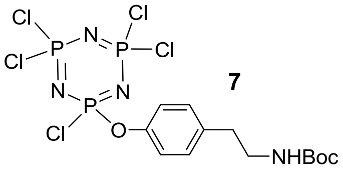

7

Synthesis of 7.

To a solution of the Boc-protected tyramine **2** (2.37 g, 10.0 mmol) in THF, Cs_2CO_3 (6.52 g, 20.0 mmol) and the core $N_3P_3Cl_6$ (4.17 g, 12 mmol) were added at rt. The reaction mixture was stirred for an additional 16 h at rt and continuously monitored by TLC. After complete reaction the salts were sedimented, the organic phase was collected, and the solvent was removed in vacuo. Pure, monofunctionalized compound **7** was obtained by column chromatography (silica gel, hexane/ethyl acetate (4:1 v:v) as eluent) as a colourless oil (2.65 g, 4.83 mmol, 48.3%). R_f = 0.54 (hexane/ethyl acetate (2:1 v:v)).

^{31}P {^1H} NMR (81.0 MHz, $CDCl_3$): δ 15.64 (t, ^2J (P,P) = 61.0 Hz, 1P, PO), 25.86 (d, ^2J (P,P) = 61.0 Hz, 2P, PCl_2); ^1H NMR (250 MHz, $CDCl_3$): δ 1.42 (s, 9H, CH_3), 2.79 (t, ^3J = 7.0 Hz, 2H, CH_2Ar), 3.35 (m, ^3J = 6.6 Hz, 2H, CH_2N), 4.55 (br s, 1H, NH), 7.20 (m, 4H, Ar*H*); ^{13}C {^1H} NMR (62.9 MHz, $CDCl_3$): δ 28.36 ($C(CH_3)_3$), 35.57 (CH_2Ar), 41.64 (CH_2N), 79.38 ($C(CH_3)_3$), 121.35 (d, ^2J (C,P) = 6.1 Hz, Ar*C*), 130.24 (d, ^2J (C,P) = 3.1 Hz, Ar*C*), 137.72 (Ar*C*), 147.80 (d, ^2J (C,P) = 10.7 Hz, Ar*C*), 155.78 (CO). MS (CI, NH_4^+, CH_2Cl_2) *m*/*z* (%): 566 (100) $[M + NH_4]^+$, 549 (17.2) $[M + H]^+$.

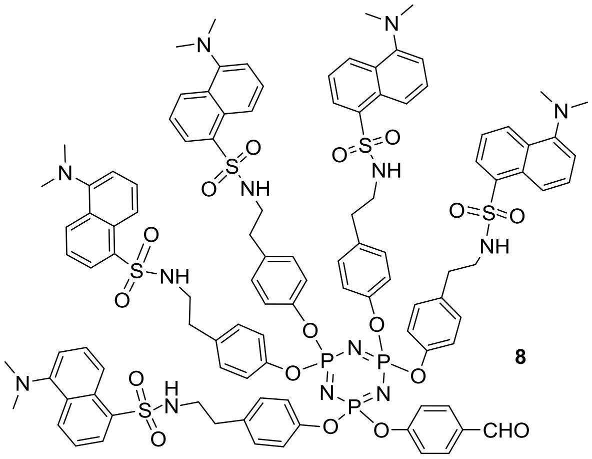

Synthesis of 8.

To a solution of the dansylated tyramine **3** (1.02 g, 2.75 mmol) in THF were added Cs_2CO_3 (1.79 g, 5.5 mmol) and the aldehyde monofunctionalized derivative **5** (217 mg, 0.50 mmol), and the mixture was stirred for 48 h at rt. After complete reaction the salts were centrifuged/sedimented, the supernatant collected, and the solvent was removed in vacuo. The fluorescent dendron **8** was obtained by column chromatography (silica gel, hexane/ethyl acetate (1:2 v:v) as eluent) as a yellow-greenish, fluorescent solid (728 mg, 346 µmol, 69.2%). R_f 0.23 (hexane/ethyl acetate (1:2 v:v)).

^{31}P {^{1}H} NMR (81.0 MHz, $CDCl_3$): δ 11.90 (m); ^{1}H NMR (250 MHz, $CDCl_3$): δ 2.62 (t, ^{3}J = 6.7 Hz, 10H, CH_2Ar), 2.86 (s, 30H, CH_3), 3.08 (s, 10H, CH_2N), 5.06 (t, ^{3}J = 6.0 Hz, 2H, NH), 5.17 (t, ^{3}J = 5.8 Hz, 3H, NH), 6.75 (m, 10H, Ar*H*), 6.81 (m, 10H, Ar*H*), 7.03 (d, ^{3}J = 8.3 Hz, 2H, Ar*H*), 7.12 (m, 5H, ArH_{Dns}), 7.46 (m, 10H, ArH_{Dns}), 7.63 (d, ^{3}J = 8.8 Hz, 2H, Ar*H*), 8.19 (m, 10H, ArH_{Dns}), 8.53 (d, ^{3}J = 8.8 Hz, 5H, ArH_{Dns}), 9.85 (s, 1H, CHO); ^{13}C {^{1}H} NMR (62.9 MHz, $CDCl_3$): δ 35.00 (CH_2Ar), 44.25 (s, CH_2N), 45.44 (CH_3), 115.28 (ArC_{Dns}), 118.88 (ArC_{Dns}), 120.88 (Ar*C*), 121.35 (Ar*C*), 123.26 (Ar*C*), 128.46 (ArC_{Dns}), 129.52 (ArC_{Dns}), 129.59 (ArC_{Dns}), 129.74 (ArC_{Dns}), 130.42 (ArC_{Dns}), 131.34 (ArC_{Dns}), 133.03 (ArC_{Dns}), 134.60 (Ar*C*), 134.75 (Ar*C*), 134.81(Ar*C*), 134.97 (Ar*C*), 149.03 (Ar*C*), 151.72 (ArC_{Dns}), 191.16 (CHO); MS (IS, positive, DMF/MeOH + HCO_2H) *m*/*z*: 2142 $[M + K]^+$, 2125 $[M + Na]^+$, 2104 $[M + H]^+$, 1870 $[M - (C_{12}H_{12}NO_2S) + H]^+$, 1769, 1636 $[M - 2(C_{12}H_{12}NO_2S) + H]^+$, 1090 $[M + 2K]^{2+}$, 1082 $[M + K + Na]^{2+}$, 1072 $[M + H + K]^{2+}$, 1052 $[M + 2H]^{2+}$; monoisotopic mass calcd for $C_{107}H_{111}N_{13}O_{17}P_3S_5^+$, 2102.60; found, 2102.75.

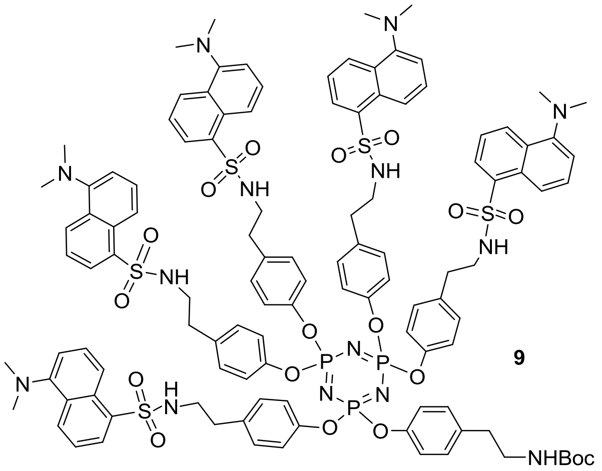

9

Synthesis of 9.

Cs_2CO_3 (2.51 g, 7.70 mmol) was added to a solution of the dansylated tyramine **3** (1.43 g, 3.85 mmol) in THF at rt. After 5 min derivative **6** (0.384 g, 0.700 mmol) was added, and the reaction mixture was stirred for 48 h at rt. After complete reaction (TLC- and ^{31}P NMR-monitored) the salts were sedimented or centrifuged off, the supernatant was collected, and the crude product was purified by column chromatography (silica gel, gradient of hexane/ethyl acetate (1:1 v:v) → (1:3 v:v) as eluent). The procedure gave the dendron **9** (0.878 g, 0.396 mmol, 56.6%) as a bright yellow solid (foam). R_f 0.38 (hexane/ethyl acetate (1:2 v:v)).

^{31}P {^{1}H} NMR (81.0 MHz, $CDCl_3$): δ 12.16 (br m); ^{1}H NMR (250 MHz, $CDCl_3$): δ 1.41 (s, 9H, $C(CH_3)_3$), 2.61 (m, 10H + 2H, CH_2Ar), 2.84 (s, 30H, $N(CH_3)_2$), 3.07 (m, 10H, CH_2N), 3.26 (m, 2H, CH_2N), 4.71 (br t, ^{3}J = 5.3 Hz, 1H, NHCO), 5.08 (br t, ^{3}J = 6.0 Hz, 5H, $NHSO_2$), 6.76 (m, 10H + 10H + 2H, Ar*H*), 6.93 (d, ^{3}J = 8.3 Hz, 2H, Ar*H*), 7.12 (m, 5H, ArH_{Dns}), 7.45 (m, 10H, ArH_{Dns}), 8.18 (m, 10H, ArH_{Dns}), 8.51 (d, ^{3}J = 8.8 Hz, 5H, ArH_{Dns}); ^{13}C {^{1}H} NMR (62.9 MHz, $CDCl_3$): δ 28.43 ($C(CH_3)_3$), 35.07 (CH_2Ar_{Dns}), 35.46 (CH_2Ar), 41.87 (CH_2N), 44.25 (CH_2N_{Dns}), 45.41 ($N(CH_3)_2$), 79.41 (*C*$(CH_3)_3$), 115.21 (Ar*C*), 118.72 (Ar*C*), 119.66 (Ar*C*), 120.95 (Ar*C*), 123.20 (Ar*C*), 128.42 (Ar*C*), 128.49 (Ar*C*), 129.52 (Ar*C*), 129.64 (Ar*C*), 129.80 (Ar*C*), 130.46 (Ar*C*), 134.60 (Ar*C*), 134.72 (Ar*C*), 135.62 (Ar*C*), 149.15 (Ar*C*), 151.91 (Ar*C*), 155.98 (CO). MS (IS, positive, DMSO/MeOH) *m*/*z*: 2256 $[M + K]^+$, 2240 $[M + Na]^+$, 2218 $[M + H]^+$; monoisotopic mass calcd for $C_{113}H_{124}N_{14}O_{18}P_3S_5^+$, 2217.70; found, 2217.75.

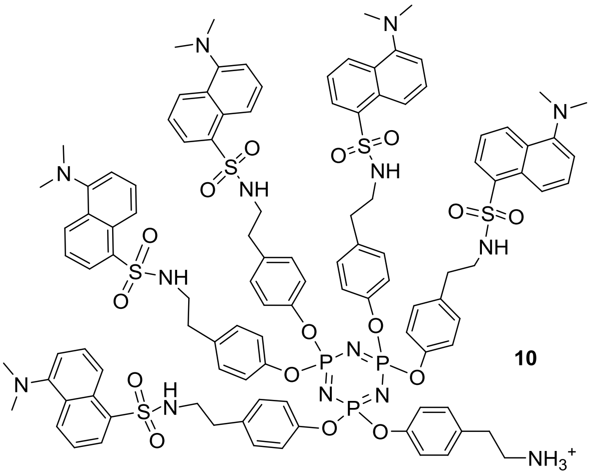

10

Synthesis of 10.

TFA (25% (v:v)) was added to a solution of the dansylated dendron **9** (222 mg, 100 µmol) in CH_2Cl_2, and the mixture was stirred for 60 min at rt. After evaporation of the solvent the procedure was repeated one more time (60 min at rt) and the mixture was monitored by ^{1}H NMR spectroscopy. After complete Boc deprotection the solvent was repeatedly removed in vacuo until the green fluorescence reappeared. The fluorescent dendron **10** (219 mg, 98.1 µmol, 98.1%) was obtained as a bright yellow solid after freeze-drying from dioxane.

^{31}P {^{1}H} NMR (81.0 MHz, CD_3OD): δ 12.30 (br m); ^{1}H NMR (500 MHz, CD_3OD): δ 2.54 (m, 6H, CH_2Ar_{Dns}), 2.60 (m, 4H, CH_2Ar_{Dns}), 2.92 (s, 12H, $N(CH_3)_2$), 2.93 (s, 18H, $N(CH_3)_2$), 2.96 (m, 2H, CH_2Ar), 3.02 (m, 10H, CH_2N), 3.17 (m, 2H, CH_2N), 6.50 (d, ^3J = 6.5 Hz, 4H, Ar*H*), 6.54 (d, ^3J = 8.5 Hz, 2H, Ar*H*), 6.63 (d, ^3J = 8.5 Hz, 4H, Ar*H*), 6.71 (d, ^3J = 8.5 Hz, 4H, Ar*H*), 6.73 (d, ^3J = 8.3 Hz, 2H, Ar*H*), 6.82 (d, ^3J = 8.5 Hz, 4H, Ar*H*), 6.88 (d, ^3J = 8.5 Hz, 2H, Ar*H*), 7.18 (d, ^3J = 8.5 Hz, 2H, Ar*H*), 7.35 (t, ^3J = 7.4 Hz, 5H, ArH_{Dns}), 7.54 (m, 10H, ArH_{Dns}), 8.15 (m, 5H, ArH_{Dns}), 8.36 (m, 5H, ArH_{Dns}), 8.46(m, 5H, ArH_{Dns}); ^{13}C {^{1}H} NMR (62.9 MHz, CD_3OD): δ 33.25 (CH_2Ar_{Dns}), 35.50 (CH_2Ar), 41.23 (CH_2N), 44.70 (CH_2N_{Dns}), 45.71 ($N(CH_3)_2$), 116.89 (Ar*C*), 121.02 (Ar*C*), 121.81 (Ar*C*), 122.09 (Ar*C*), 124.72 (Ar*C*), 128.38 (Ar*C*), 129.16 (Ar*C*), 129.32 (Ar*C*), 129.69 (Ar*C*), 130.16 (Ar*C*), 130.45 (Ar*C*), 136.14 (Ar*C*), 136.27 (Ar*C*), 136.80 (Ar*C*), 148.79 (Ar*C*), 149.325 (Ar*C*), 150.26 (Ar*C*); MS (IS, positive, MeOH) *m*/*z*: 2140 $[M + Na]^+$, 2118 $[M + H]^+$, 1885 $[M - Dns + H]^+$, 1059 $[M + 2H]^{2+}$; monoisotopic mass calcd for $C_{108}H_{116}N_{14}O_{16}P_3S_5^+$, 2117.65; found, 2117.75.

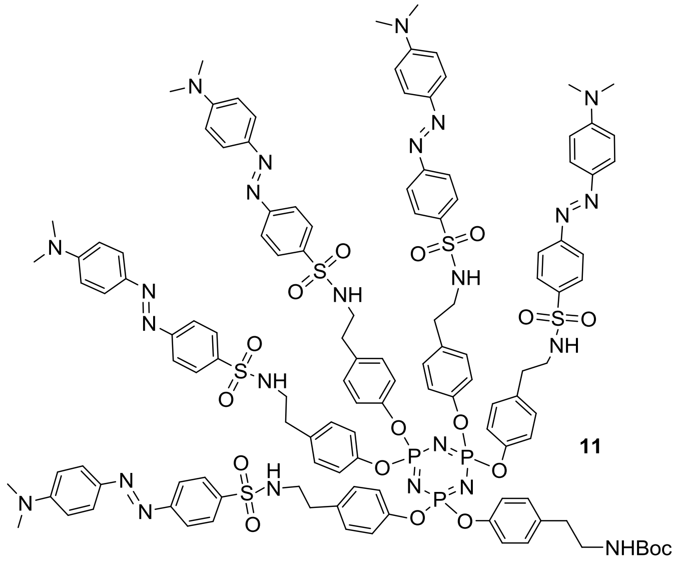

11

Synthesis of 11.

To a solution of the Boc-tyramine monofunctionalized core **7** (165 mg, 300 µmol) in THF, Cs_2CO_3 (1.08 g, 3.30 mmol) and the dabsylated tyramine **4** (700 mg, 1.65 mmol) were added. The reaction mixture was stirred for 48 h at rt. After complete reaction (TLC- and ^{31}P NMR-monitored) the salts were sedimented or centrifuged off, the supernatant was collected, and the solvent was removed in vacuo. Purification of the crude product by column chromatography (silica gel, gradient of ethyl acetate in hexane 50% → 100% (v:v) as eluent) gave the dabsylated AB_5 dendron **11** (324 mg, 130 mmol, 43.3%) as a red solid. R_f 0.18 (ethyl acetate/hexane (2:1 v:v)).

^{31}P {^{1}H} NMR (81.0 MHz, acetone-d_6): δ 12.38 (br s); ^{1}H NMR (250 MHz, acetone-d_6): δ 1.38 (s, 9H, $C(CH_3)_3$), 2.75 (m, 10H, CH_2Ar_{Dbs}), 2.88 (m, 2H, CH_2Ar), 3.08 (s, 30H, $N(CH_3)_3$), 3.15 (m, 10H, CH_2N), 3.23 (m, 2H, CH_2N), 6.61 (m, 2H, Ar*H*), 6.80 (m, 10H + 10H + 2H, Ar*H*), 7.03 (m, 10H, Ar*H*), 7.85 (m, 10H, Ar*H*), 7.92 (m, 10H + 10H, Ar*H*); ^{13}C {^{1}H} NMR (62.9 MHz, acetone-d_6): δ 29.34 ($C(CH_3)_3$), 35.72 (CH_2Ar_{Dbs}), 35.96 (CH_2Ar), 40.20 ($N(CH_3)_2$), 42.54 (CH_2N), 45.17 (CH_2N_{Dbs}), 78.69 ($C(CH_3)_3$), 112.25 (Ar*C*), 121.49 (Ar*C*), 123.11 (Ar*C*), 126.29 (Ar*C*), 128.80 (Ar*C*), 130.59 (Ar*C*), 136.28 (Ar*C*), 137.06 (Ar*C*), 141.24 (Ar*C*), 144.07 (Ar*C*), 149.85 (Ar*C*), 154.15 (Ar*C*), 155.94 (Ar*C*), 156.5 (CO); MS (IS, positive, CHO_2H) *m*/*z*: 2888 $[M + H]^+$, 1244 $[M + 2H]^{2+}$; monoisotopic mass calcd for $C_{123}H_{134}N_{24}O_{18}P_3S_5^+$, 2487.81; found, 2487.75.

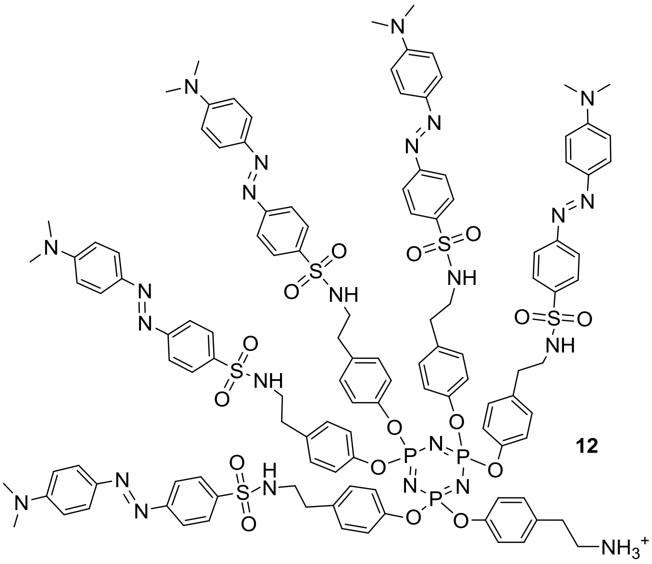

Synthesis of 12.

To a solution of the dabsylated AB_5 dendron **11** (100 mg, 40.2 µmol) in CH_2Cl_2, TFA (10% (v:v)) was added, and the reaction mixture was stirred for 60 min at rt. After one more deprotection cycle with 10% TFA (v:v) in CH_2Cl_2 (60 min, rt), the solvent was repeatedly removed in vacuo, and pure **12** (99 mg, 39.6 µmol, 98.5%) was obtained as a deep-red solid after freeze-drying from dioxane.

^{31}P {^{1}H} NMR (81.0 MHz, acetone-d_6): δ 12.36 (m); ^{1}H NMR (250 MHz, acetone-d_6): δ 2.78 (m, 10H, CH_2Ar_{Dbs}), 3.11 (s, 18H, $N(CH_3)_3$), 3.12 (s, 12H, $N(CH_3)_3$), 3.16 (m, 10H, CH_2N_{Dbs}), 3.18 (m, 2H, CH_2Ar), 4.07 (m, 2H, CH_2N), 6.83 (m, 10H + 10H + 2H, Ar*H*), 7.05 (m, 10H, Ar*H*), 7.23 (d, ^{3}J = 8.4 Hz, 2H, Ar*H*), 7.85 (m, 10H, Ar*H*), 7.91 (m, 10H + 10H, Ar*H*); ^{13}C {^{1}H} NMR (62.9 MHz, acetone-d_6): δ 33.40 (CH_2Ar_{Dbs}), 35.62 ($N(CH_3)_2$), 35.69 ($N(CH_3)_2$), 40.26 (CH_2Ar), 45.11 (CH_2N), 49.52 (CH_2N_{Dbs}), 112.41 (Ar*C*), 121.46 (Ar*C*), 122.02 (Ar*C*), 123.02 (Ar*C*), 126.40 (Ar*C*), 128.81 (Ar*C*), 130.62 (Ar*C*), 130.94 (Ar*C*), 134.61 (Ar*C*), 136.39 (Ar*C*), 141.12 (Ar*C*), 141.21 (Ar*C*), 144.03 (Ar*C*), 149.85 (Ar*C*), 154.23 (Ar*C*), 155.76 (Ar*C*); MS (IS, positive, MeOH) *m*/*z*: 2388 $[M + H]^{+}$, 1205 [M + H + Na], 1194 $[M + 2H]^{2+}$; monoisotopic mass calcd for $C_{118}H_{126}N_{24}O_{16}P_3S_5^{+}$, 2387.76; found, 2387.59.

Determination of fluorescence quantum yield

UV–vis spectra were observed with a Perkin Elmer Lambda35 UV–vis spectrometer. For the measurement of the emission and excitation spectra a Horiba Jobin Yvon FluoroMax®-4 spectrofluorometer was used. The fluorescence quantum yield Φ of compounds was measured in 1,4-dioxane with quinine sulfate in 0.5 M aqueous solution of H_2SO_4 as standard (Φ = 0.546) [1]. The excitation wavelength chosen was 347 nm. The following equation was used to determine the fluorescence quantum yield (Φ):

$$\Phi_{sample} = \Phi_{standard} * \frac{Abs^{sample}}{Abs^{standard}} * \frac{A_{If}^{standard}}{A_{If}^{sample}} * \frac{n_{standard}^{2}}{n_{sample}^{2}}$$

where, $A_{If}^{standard}$ and A_{If}^{sample} correspond to the integrated areas under the corrected emission spectra, Abs^{sample} and $Abs^{standard}$ are the sample and standard absorbance

at 347 nm, and n_{sample} and $n_{standard}$ are the solvent refractive index. The absorbance was less than 0.05 at the excitation wavelength to minimize the self-absorption effect. The concentration of solutions was adjusted to $1 \cdot 10^{-6}$ $mol \cdot L^{-1}$ and the absorption, emission and excitation spectra were recorded. All measurements were carried out at least two times, and gave identical results (+/-3%).

[1] Melhuish W. H. J., *Phys. Chem.* **1961**, *65*, 229–235.
