# Supplementary material for: Synthesis of dye/fluorescent functionalized dendrons based on cyclotriphosphazene
Source: Beilstein J Org Chem. 2011 Nov 28;7:1577–83. doi: 10.3762/bjoc.7.186 (PMC3252861; doi:10.3762/bjoc.7.186)

Supporting Information

for

Synthesis of dye/fluorescent functionalized dendrons based on cyclotriphosphazene

Aurélien Hameau[1,2], Sabine Fuchs[1,2], Régis Laurent[1,2], Jean-Pierre Majoral*[1,2] and Anne-Marie Caminade*[1,2]

Address: [1]CNRS, LCC (Laboratoire de Chimie de Coordination), 205, route de Narbonne, BP 44099, F-31077 Toulouse, France and [2]Université de Toulouse; UPS, INPT, LCC, F-31077 Toulouse, France

Email: Anne-Marie Caminade - caminade@lcc-toulouse.fr; Jean-Pierre Majoral - majoral@lcc-toulouse.fr

* Corresponding author

Dedicated to the memory of our friend and former PhD student Dr Yiqian Wei who regrettably passed away on February 6th, 2011.

Spectral details

$^{31}P\ \{^{1}H\}$ NMR spectrum of dendron **8**

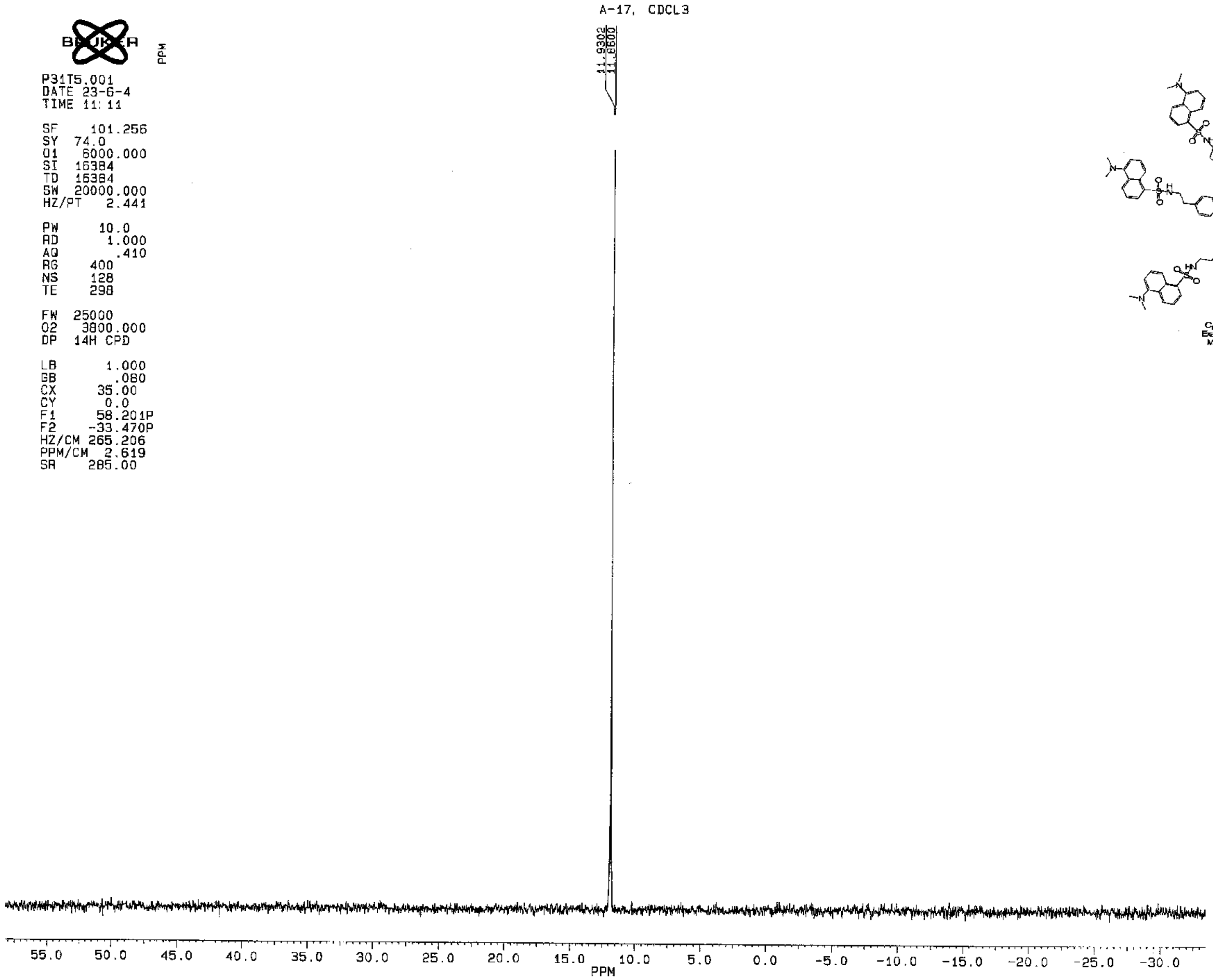

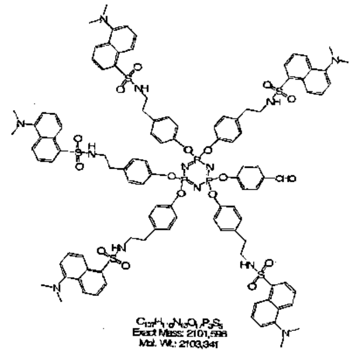

^{1}H NMR spectrum of dendron **8**

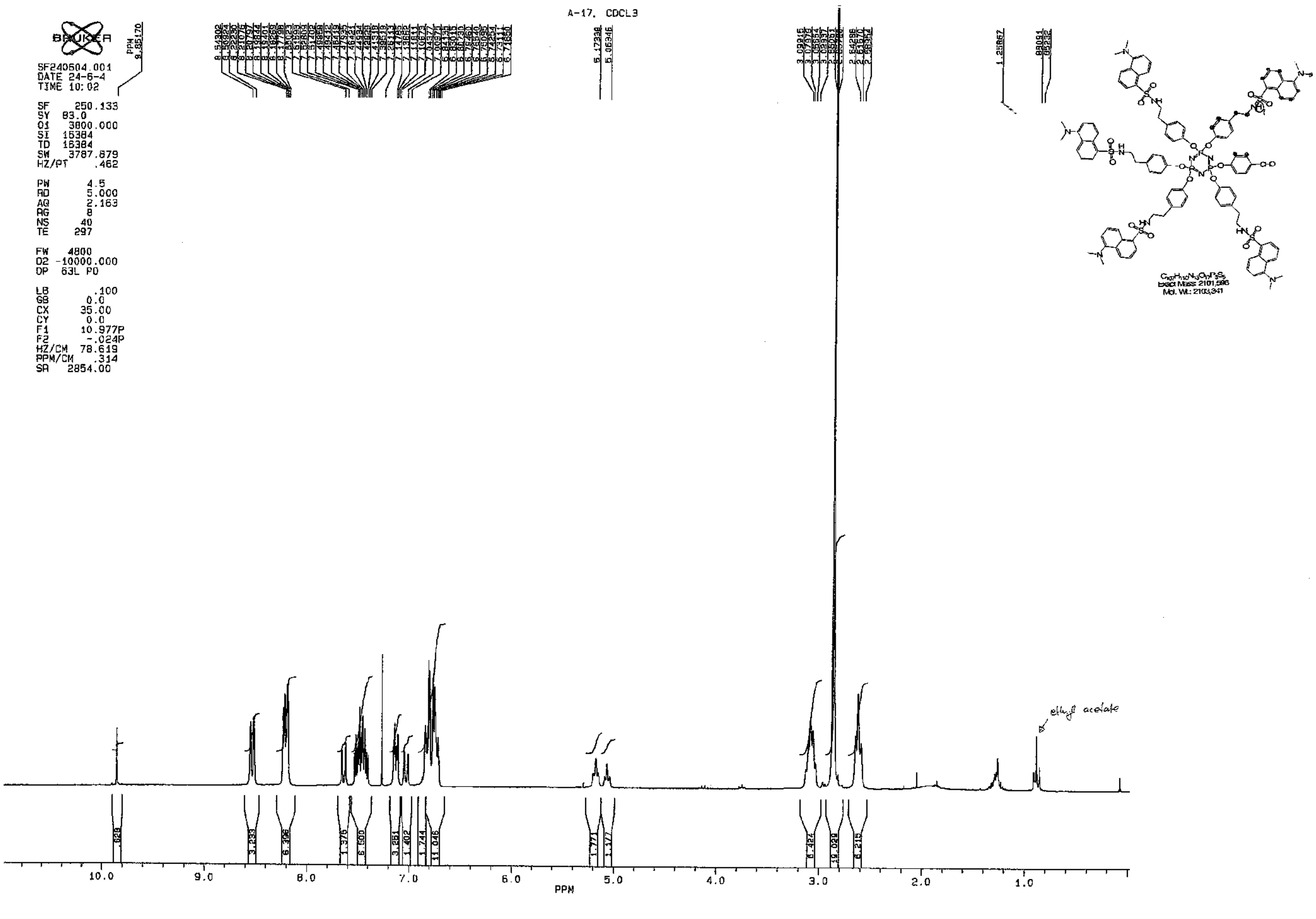

^{13}C {^{1}H} NMR spectrum of dendron **8**

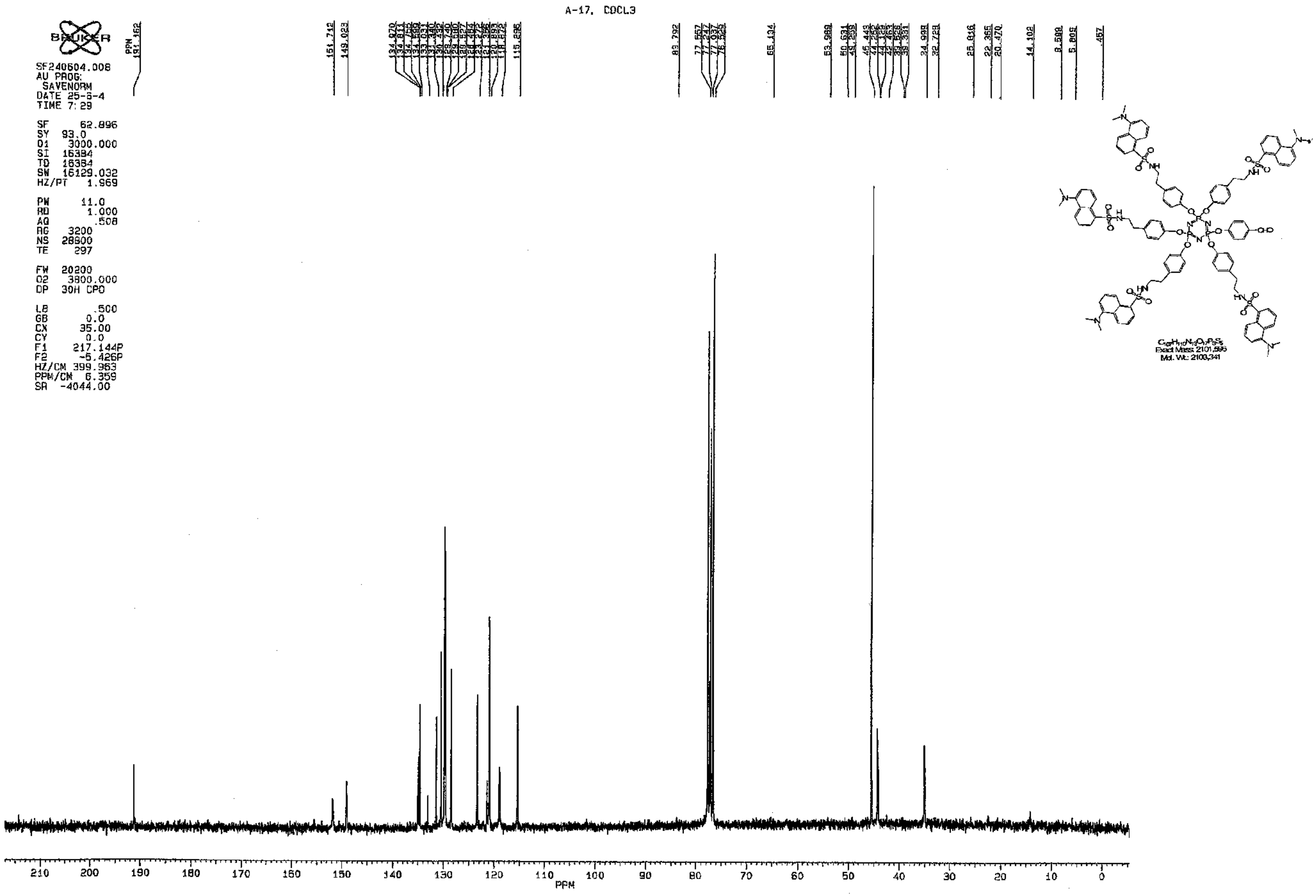

Mass spectrum (ion spray) of dendron **8**

Service Commun de Spectrométrie de Masse FR2599
Université Paul Sabatier, 118 route de Narbonne
31062 Toulouse Cedex 04

Acq. Time: 10:56
Acq. Date: Wednesday, July 21, 2004

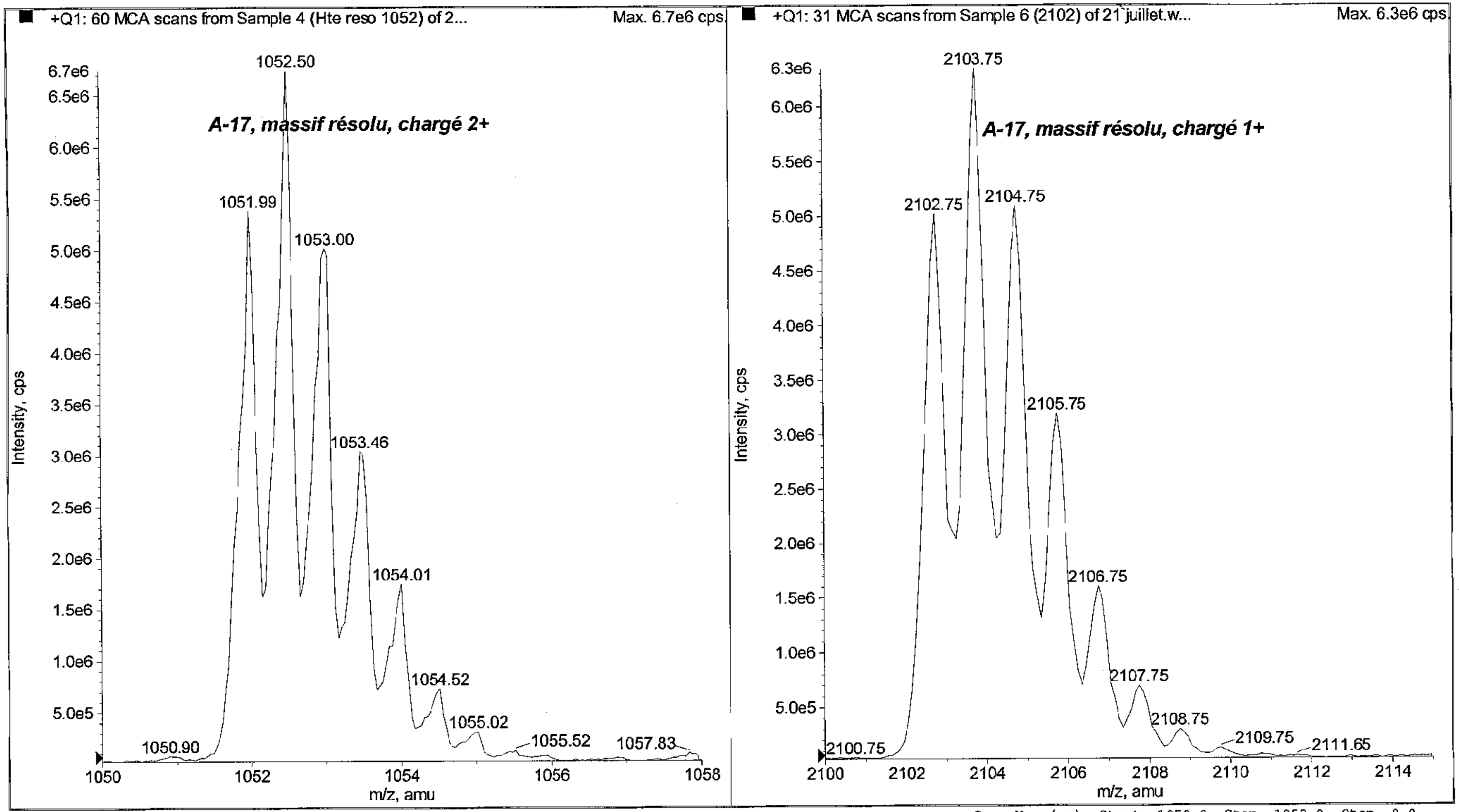

Sample Name: Hte reso 1052

Scan Mass(es): Start: 1050.0, Stop: 1058.0, Step: 0.0

Polarity/Scan Type: Positive Q1 MS
Collision Energy: N/A

^{31}P {^{1}H} NMR spectrum of dendron **9**

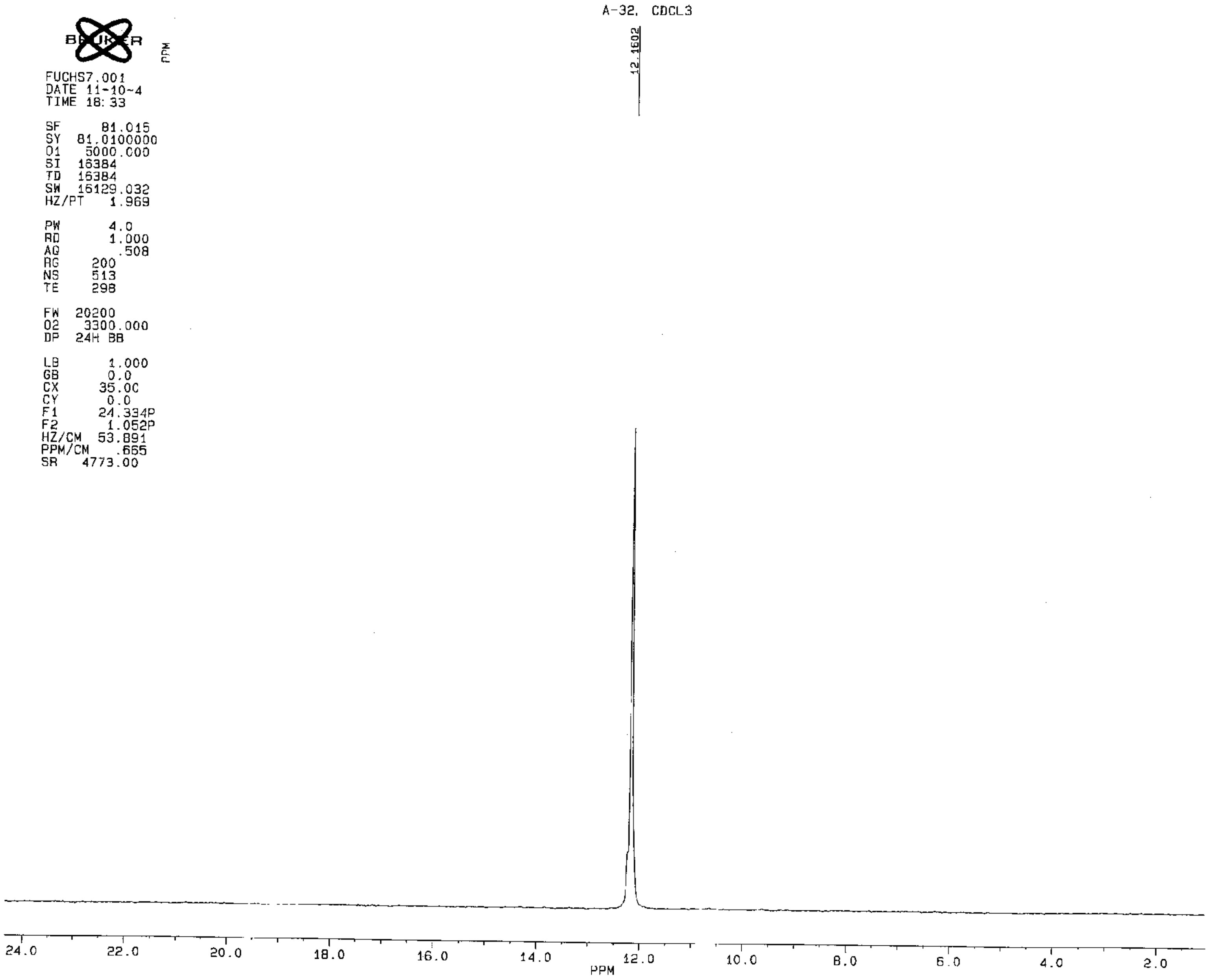

^{1}H NMR spectrum of dendron **9**

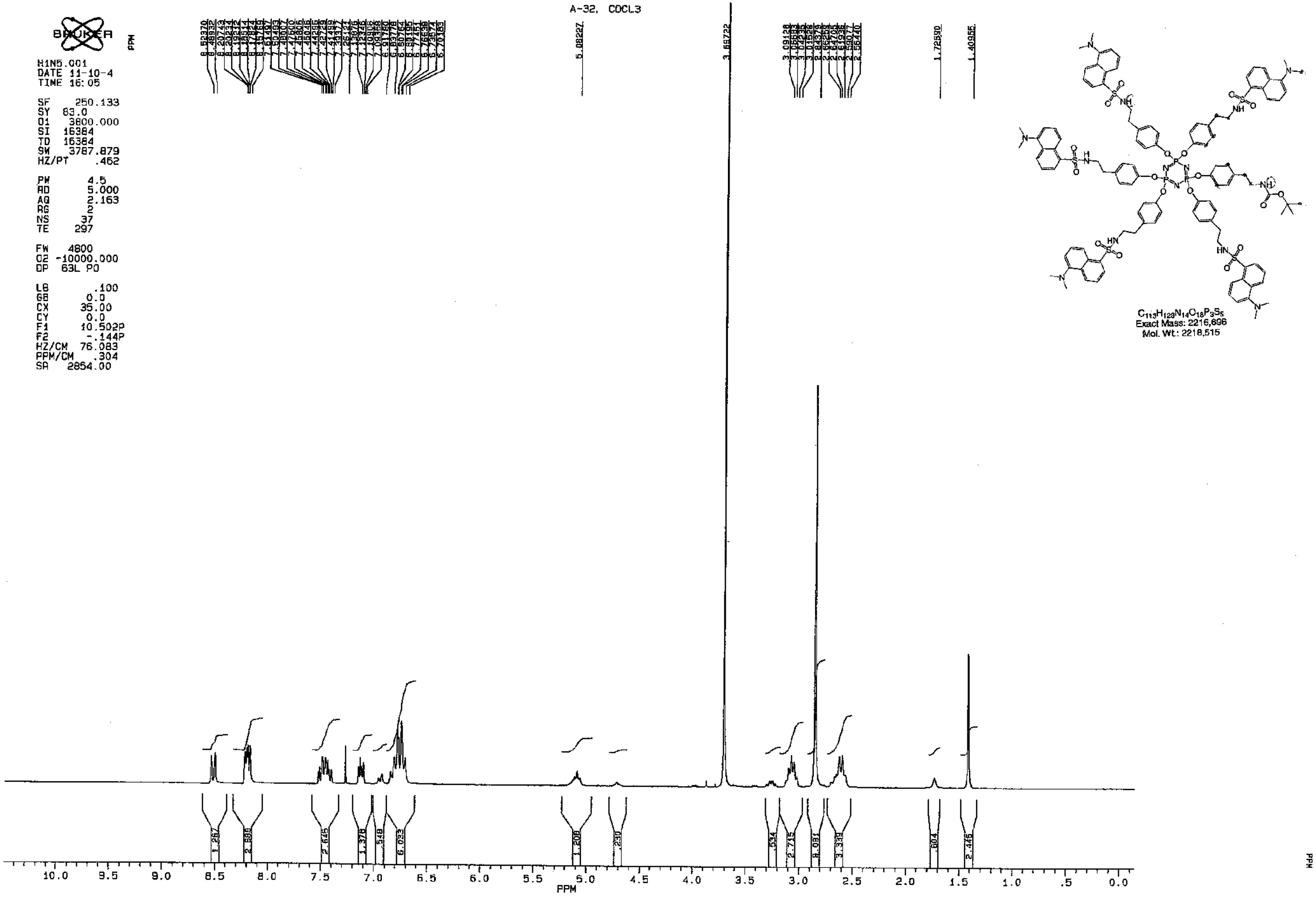

$^{13}C\ \{^{1}H\}$ NMR spectrum of dendron **9**

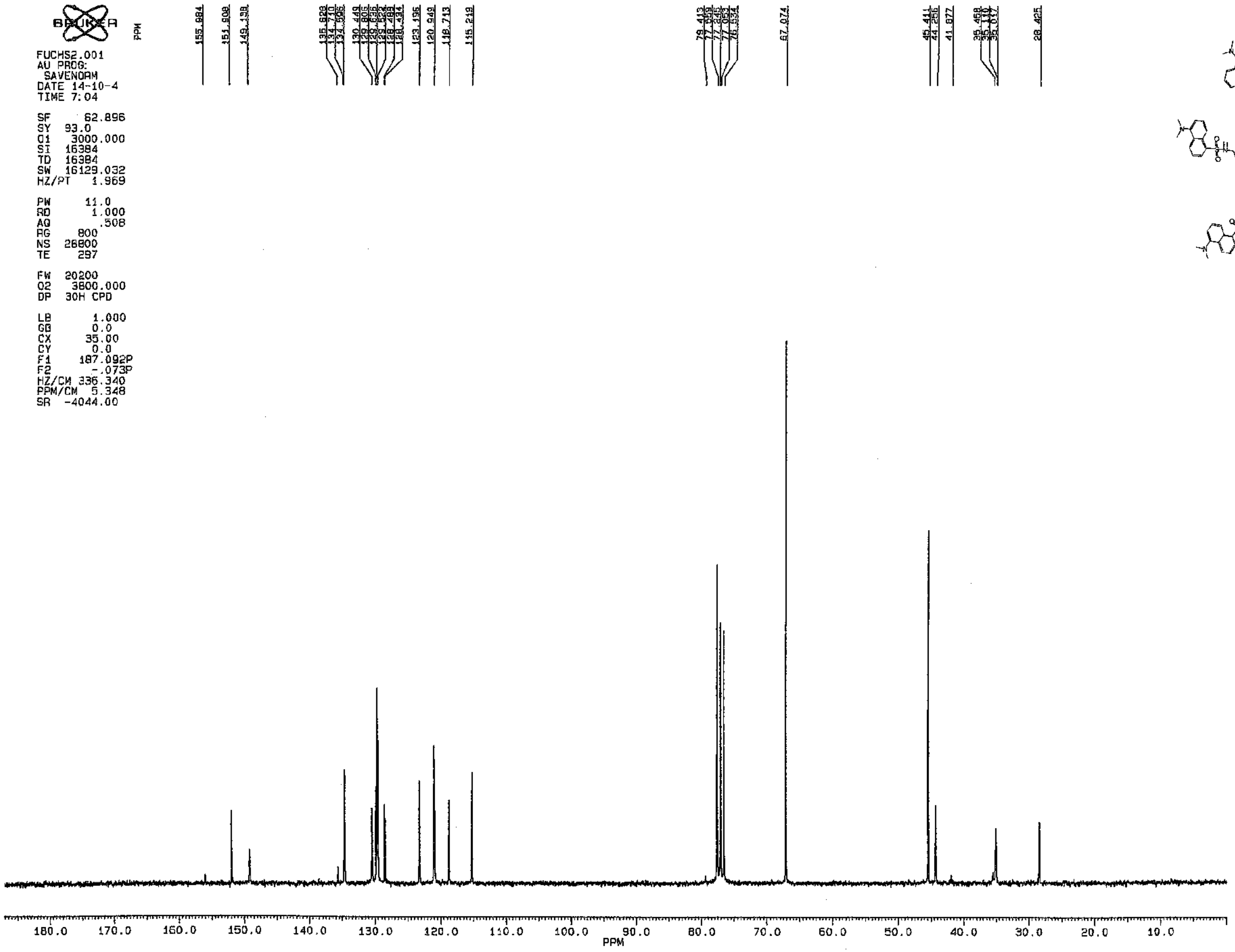

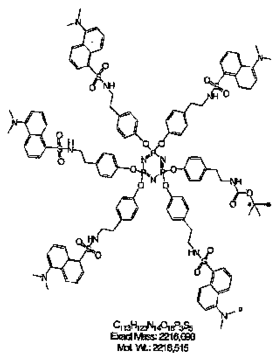

Mass spectrum (ion spray) of dendron **9**

Service Commun de Spectrométrie de Masse FR2599
Université Paul Sabatier, 118 route de Narbonne
31062 Toulouse Cedex 04

Acq. Time: 10:00
Acq. Date: Wednesday, October 20, 2004

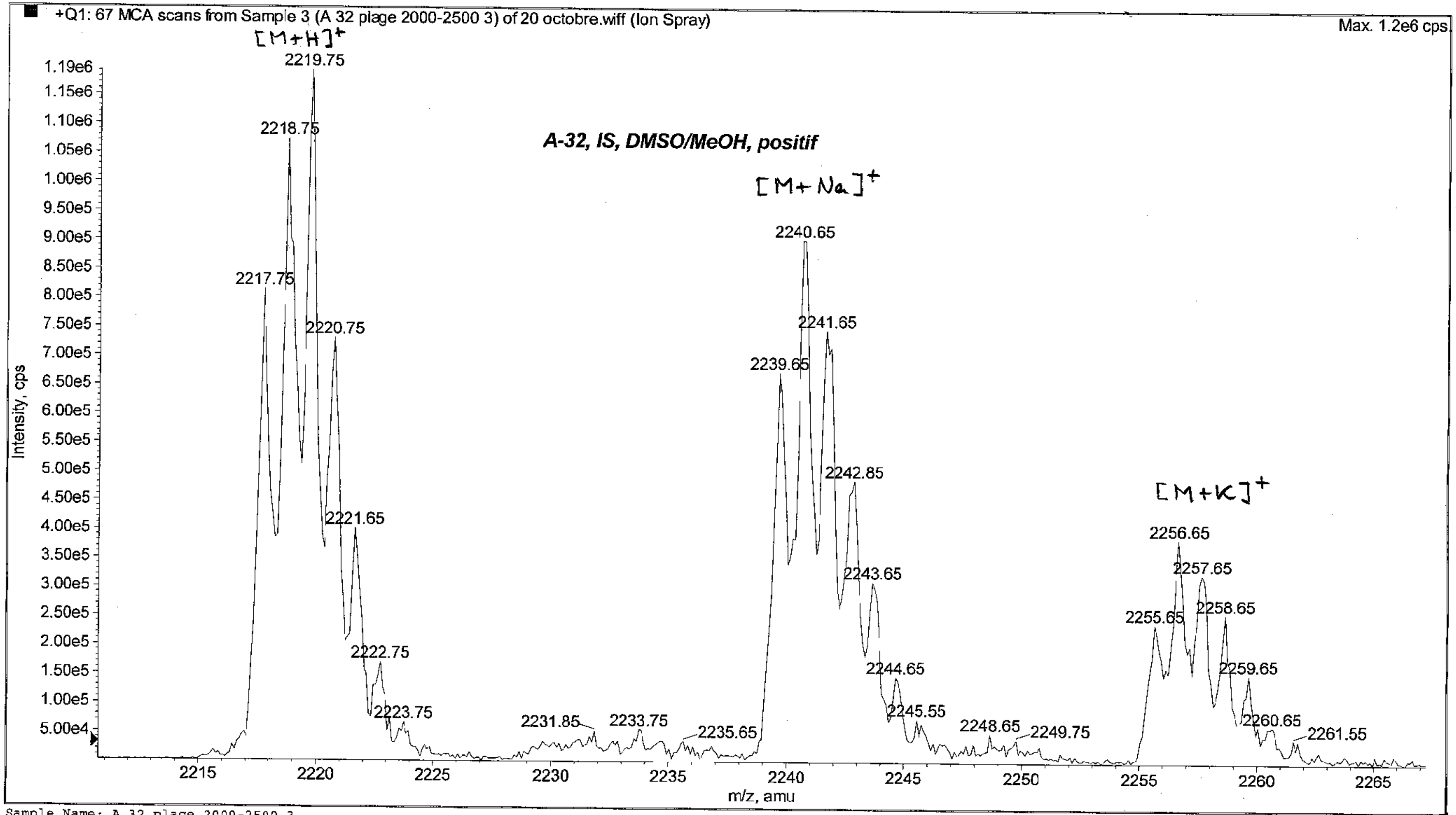

Sample Name: A 32 plage 2000-2500 3

Scan Mass(es): Start: 2200.0, Stop: 2300.0, Step: 0.1

Polarity/Scan Type: Positive Q1 MS
Collision Energy: N/A

^{31}P {^{1}H} NMR spectrum of dendron **10**

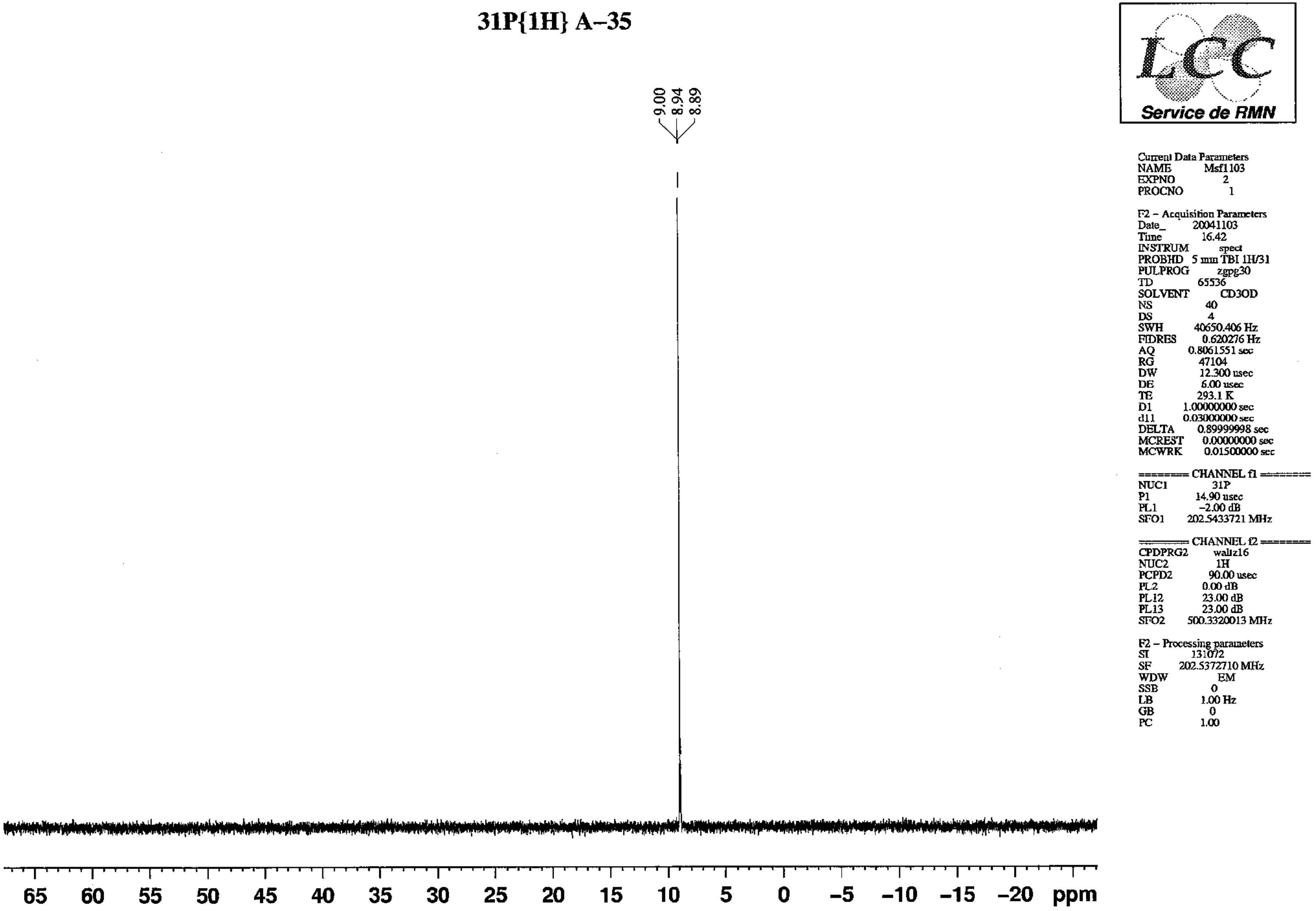

^{1}H NMR spectrum of dendron **10**

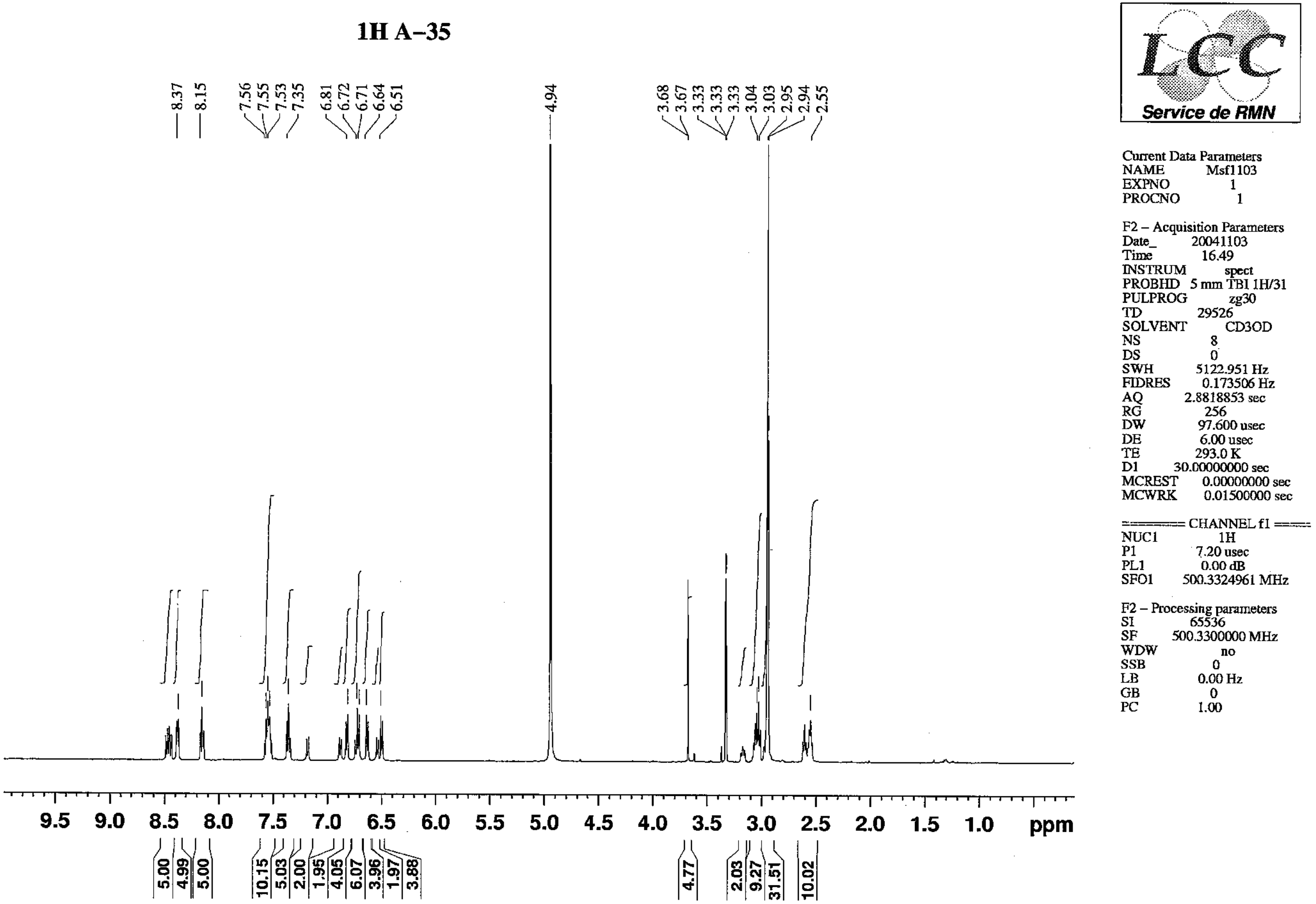

^{13}C {^{1}H} NMR spectrum of dendron **10**

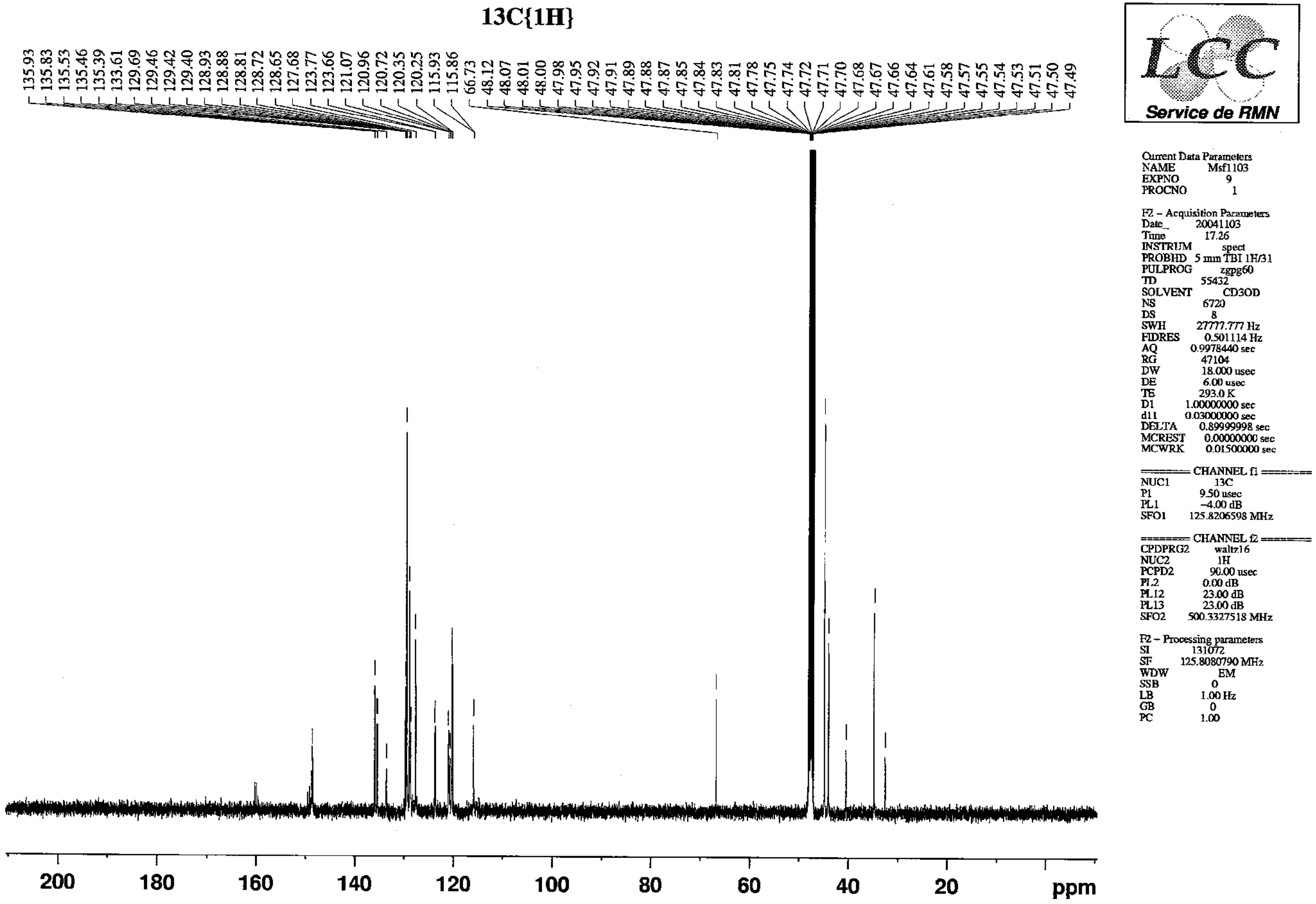

HMQC ^{1}H-^{13}C {^{31}P} NMR spectrum of dendron **10**

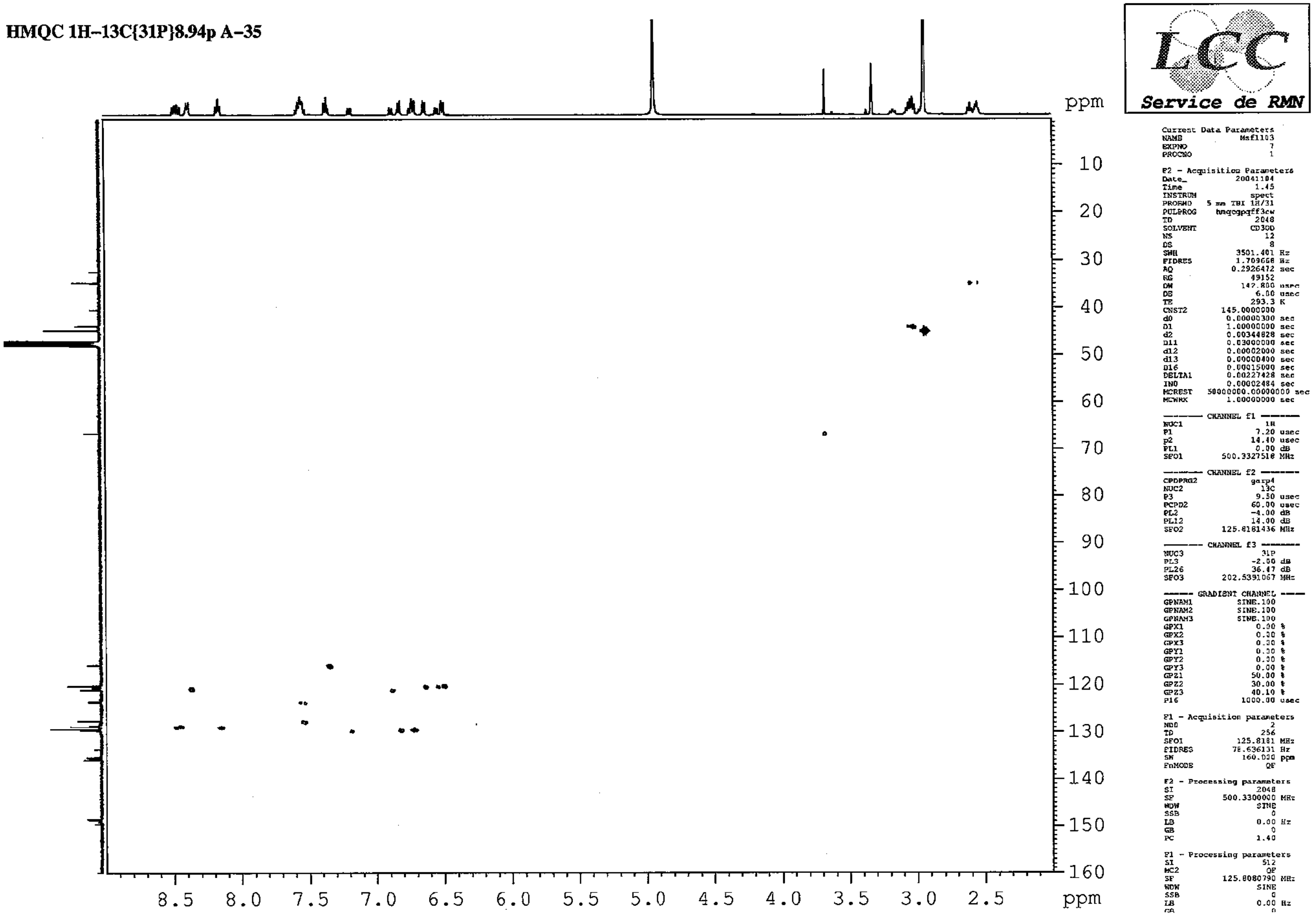

Mass spectrum (ion spray) of dendron **10**

Service Commun de Spectrométrie de Masse FR2599
Université Paul Sabatier, 118 route de Narbonne
31062 Toulouse Cedex 04

Acq. Time: 17:03
Acq. Date: Tuesday, October 19, 2004

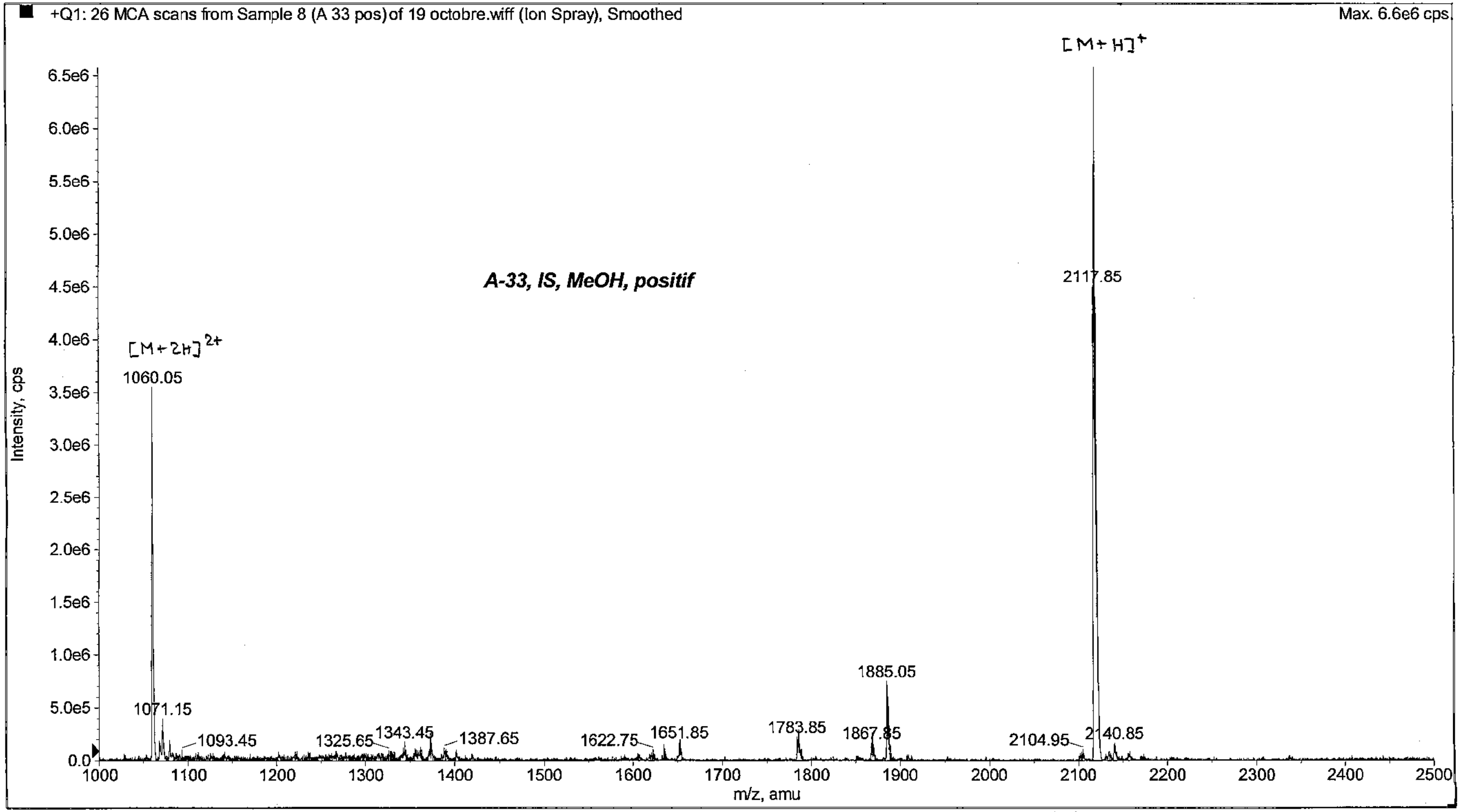

Sample Name: A 33 pos

Scan Mass(es): Start: 1000.0, Stop: 2500.0, Step: 0.1

Polarity/Scan Type: Positive Q1 MS
Collision Energy: N/A

^{31}P {^{1}H} NMR spectrum of dendron **11**

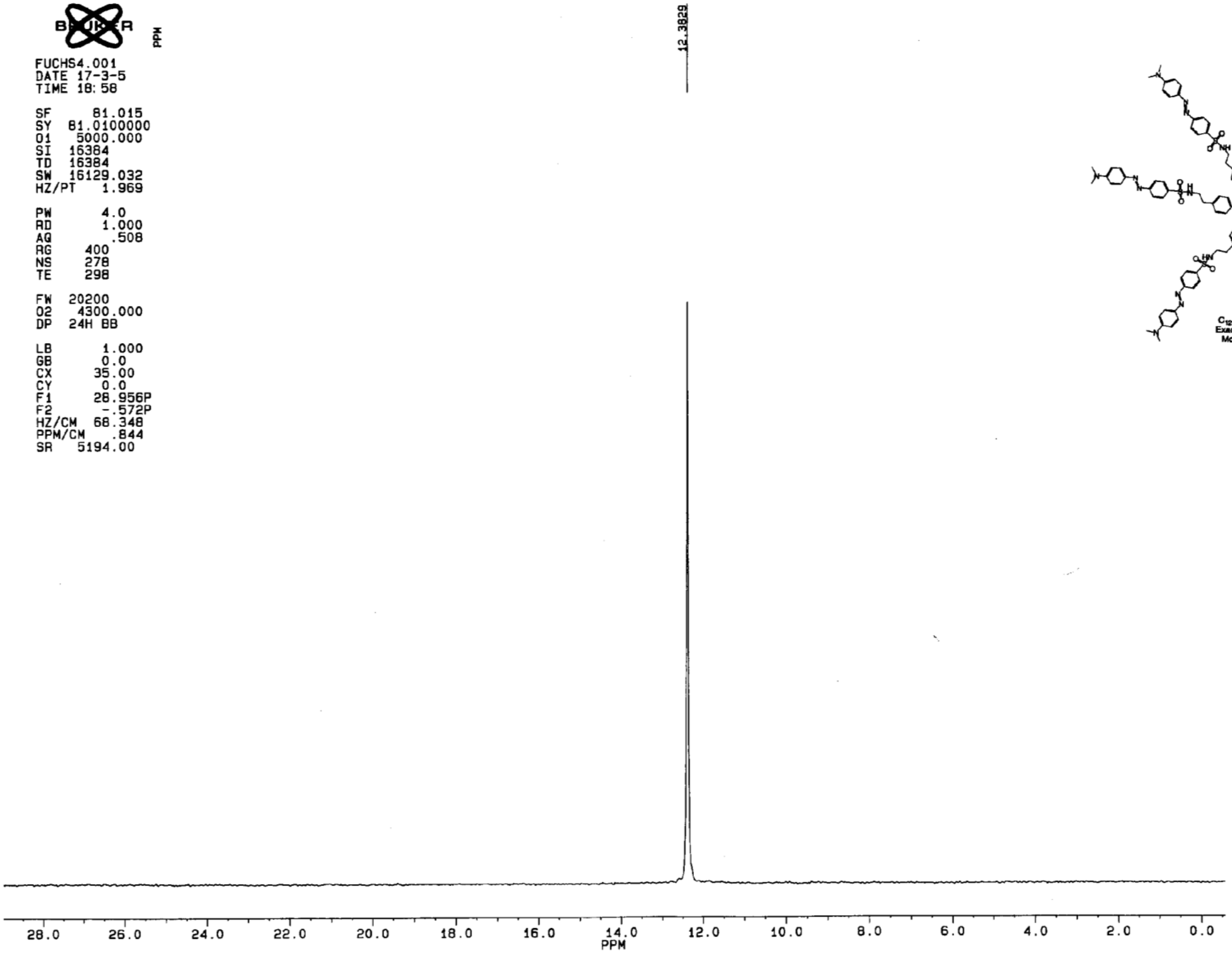

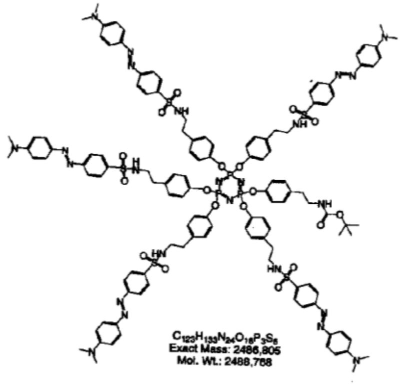

^{1}H NMR spectrum of dendron **11**

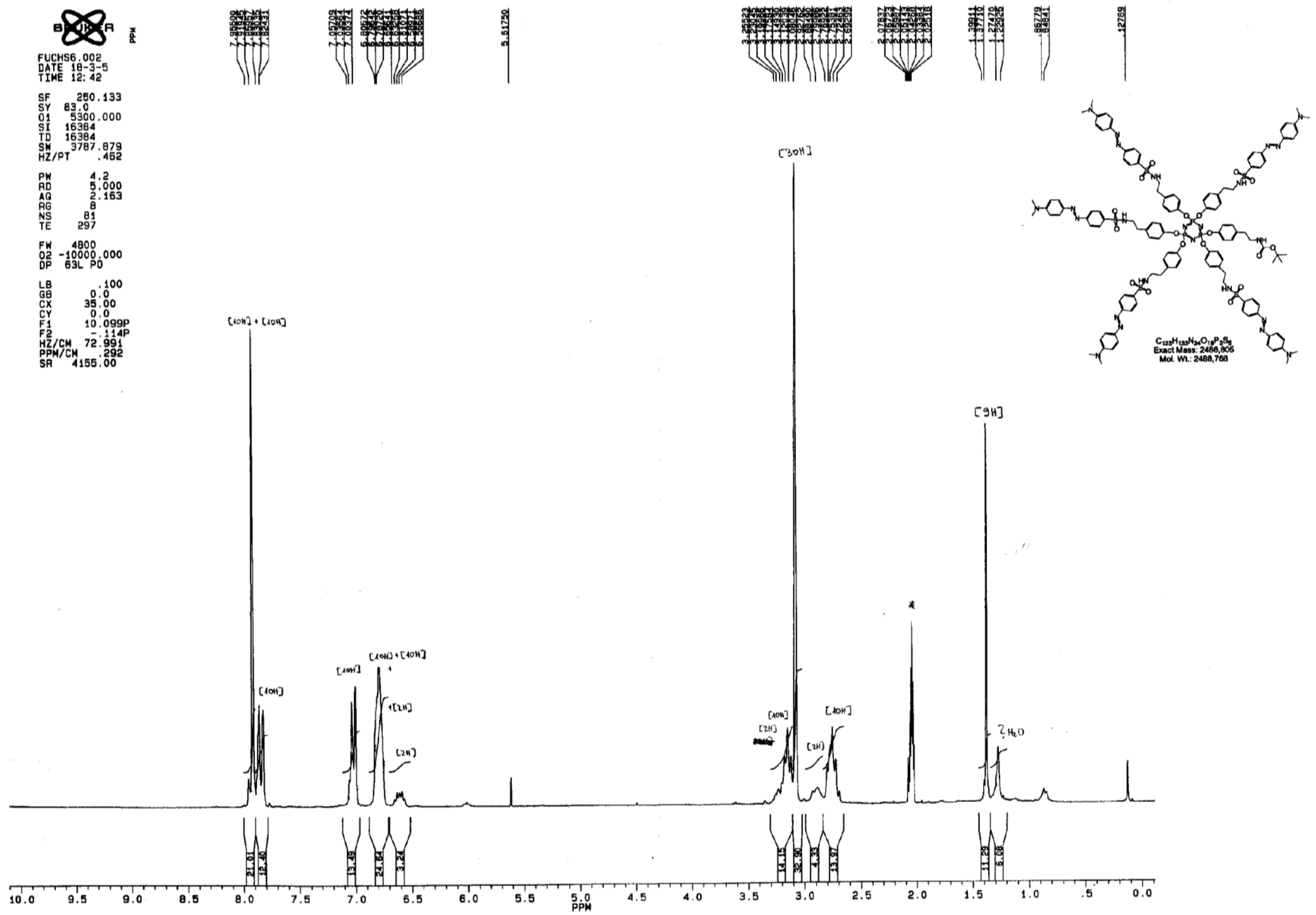

^{13}C {^{1}H} NMR spectrum of dendron **11**

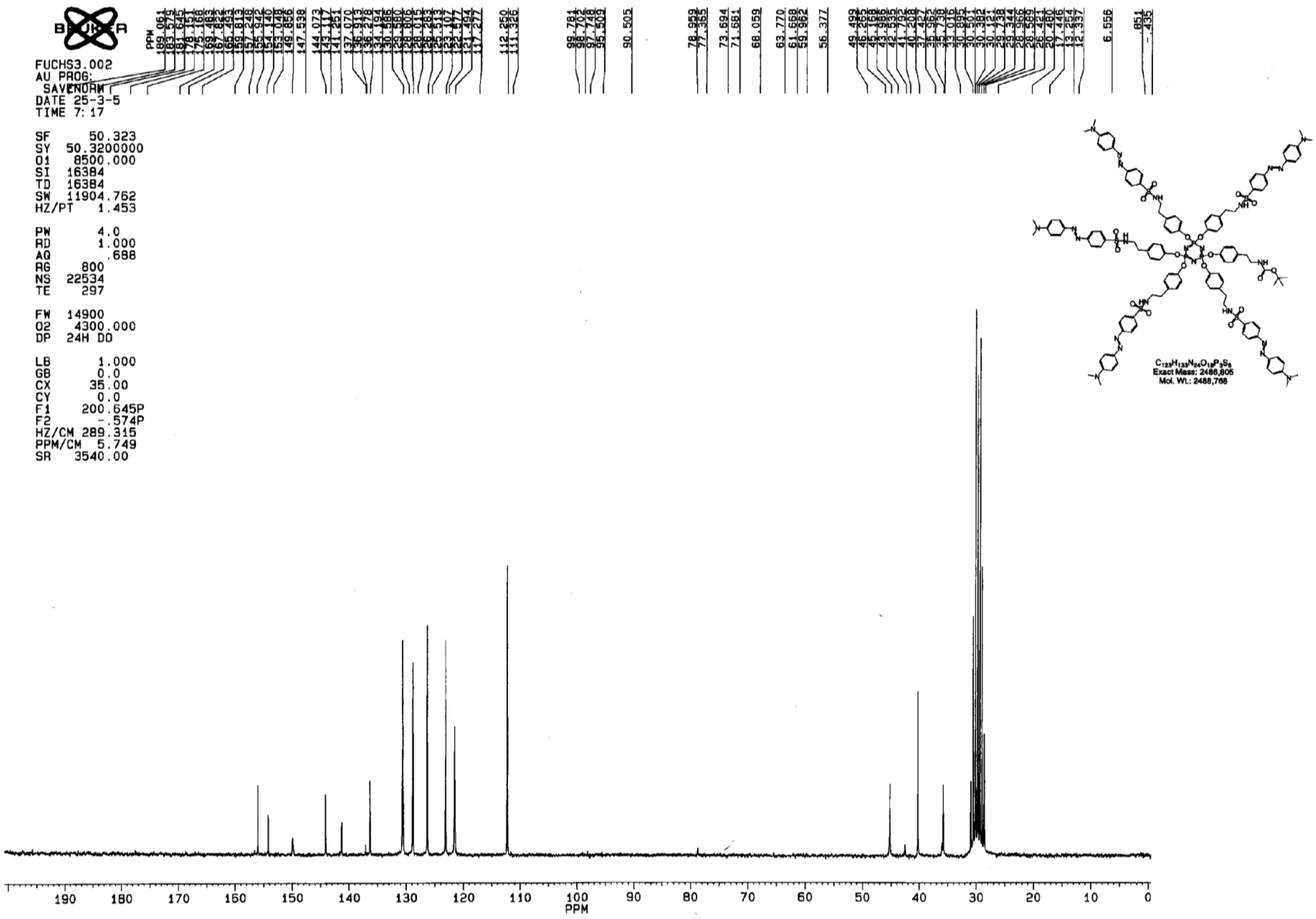

Mass spectrum (ion spray) of dendron **11**

Service Commun de Spectrométrie de Masse FR2599
Université Paul Sabatier, 118 route de Narbonne
31062 Toulouse Cedex 04

API 365

Acq. Date: Wednesday, March 23, 2005
Acq. Time: 08:52

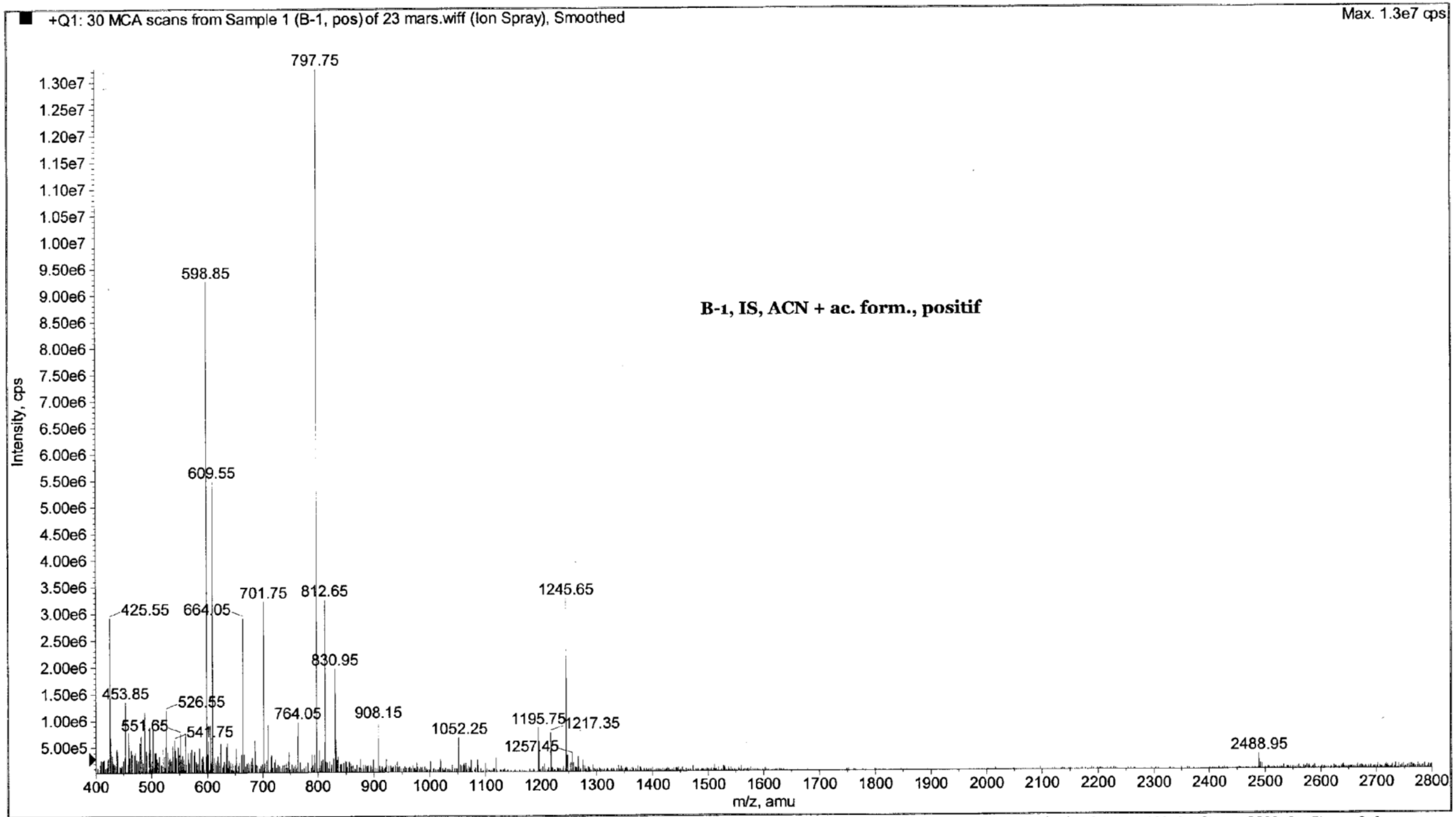

Sample Name: B-1, pos

Polarity/Scan Type: Positive Q1 MS

Scan Mass(es): Start: 400.0, Stop: 2800.0, Step: 0.1

Collision Energy: N/A

^{31}P {^{1}H} NMR spectrum of dendron **12**

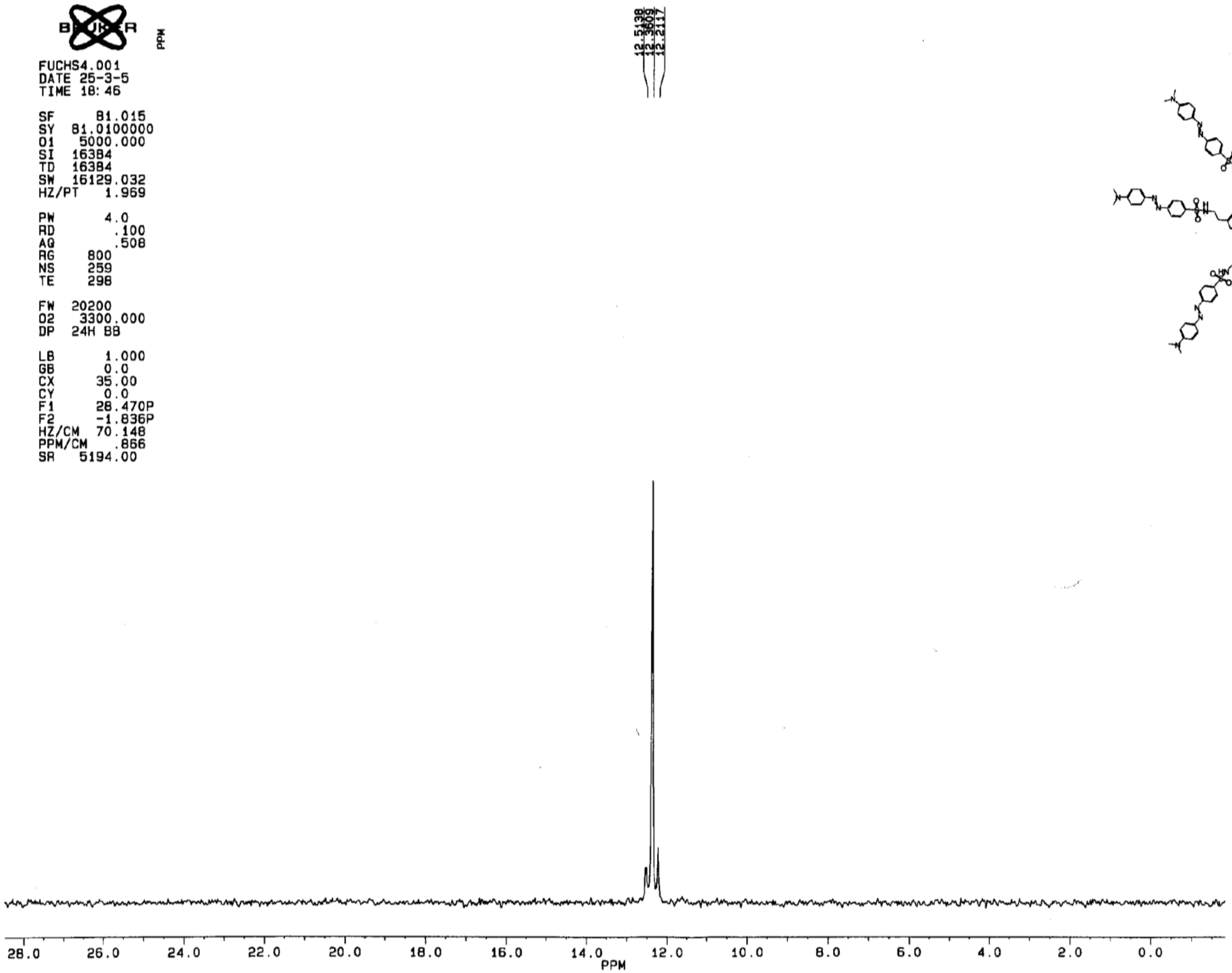

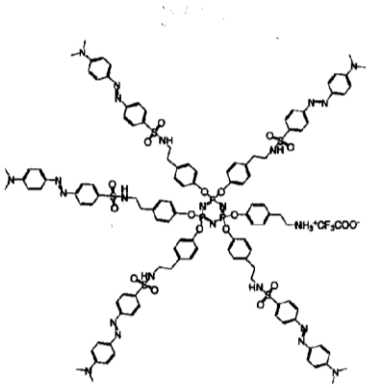

$C_{120}H_{128}F_3N_{24}O_{18}P_3S_5$
Exact Mass: 2500,745
Mol. Wt.: 2502,678

^{1}H NMR spectrum of dendron **12**

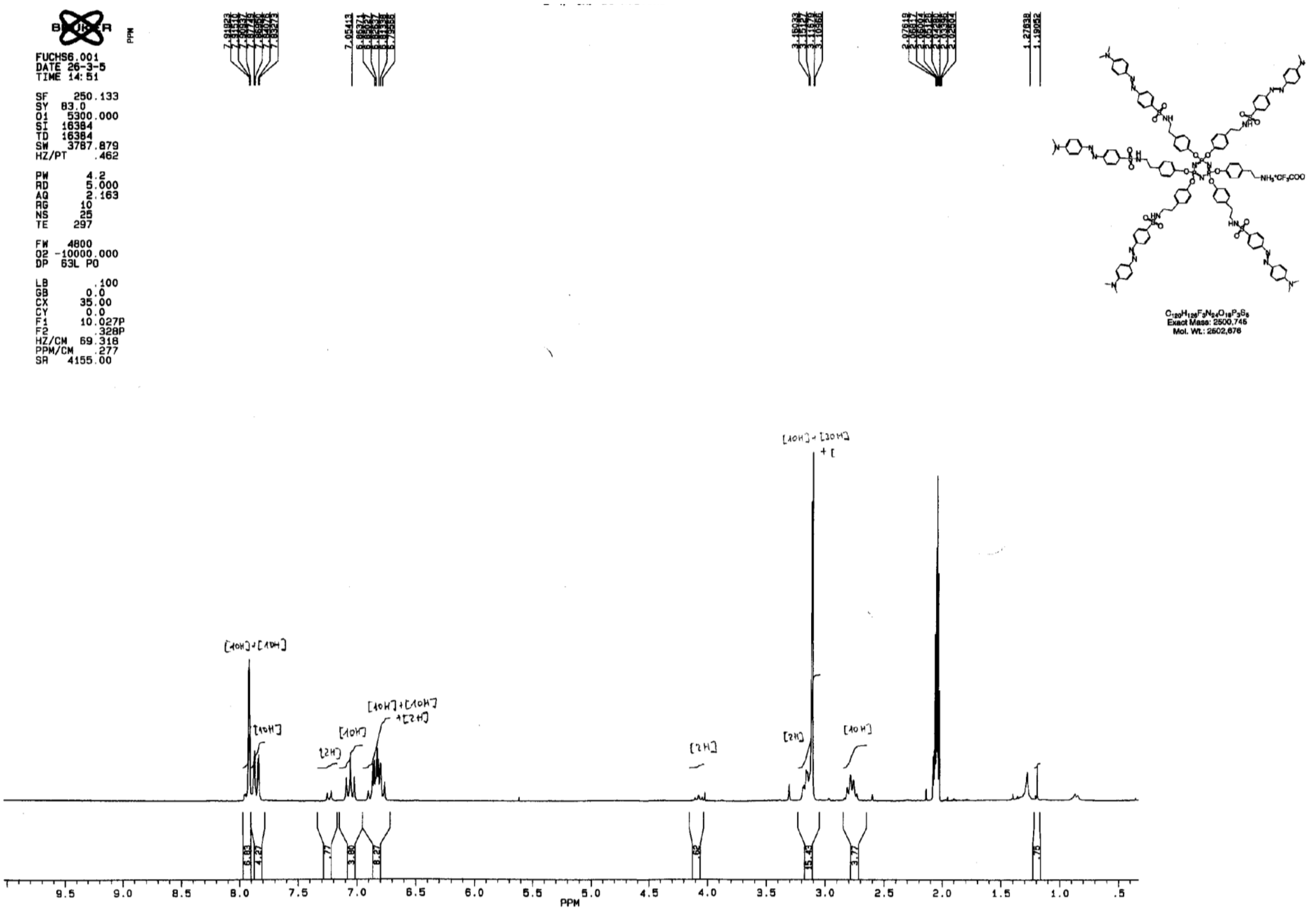

^{13}C {^{1}H} NMR spectrum of dendron **12**

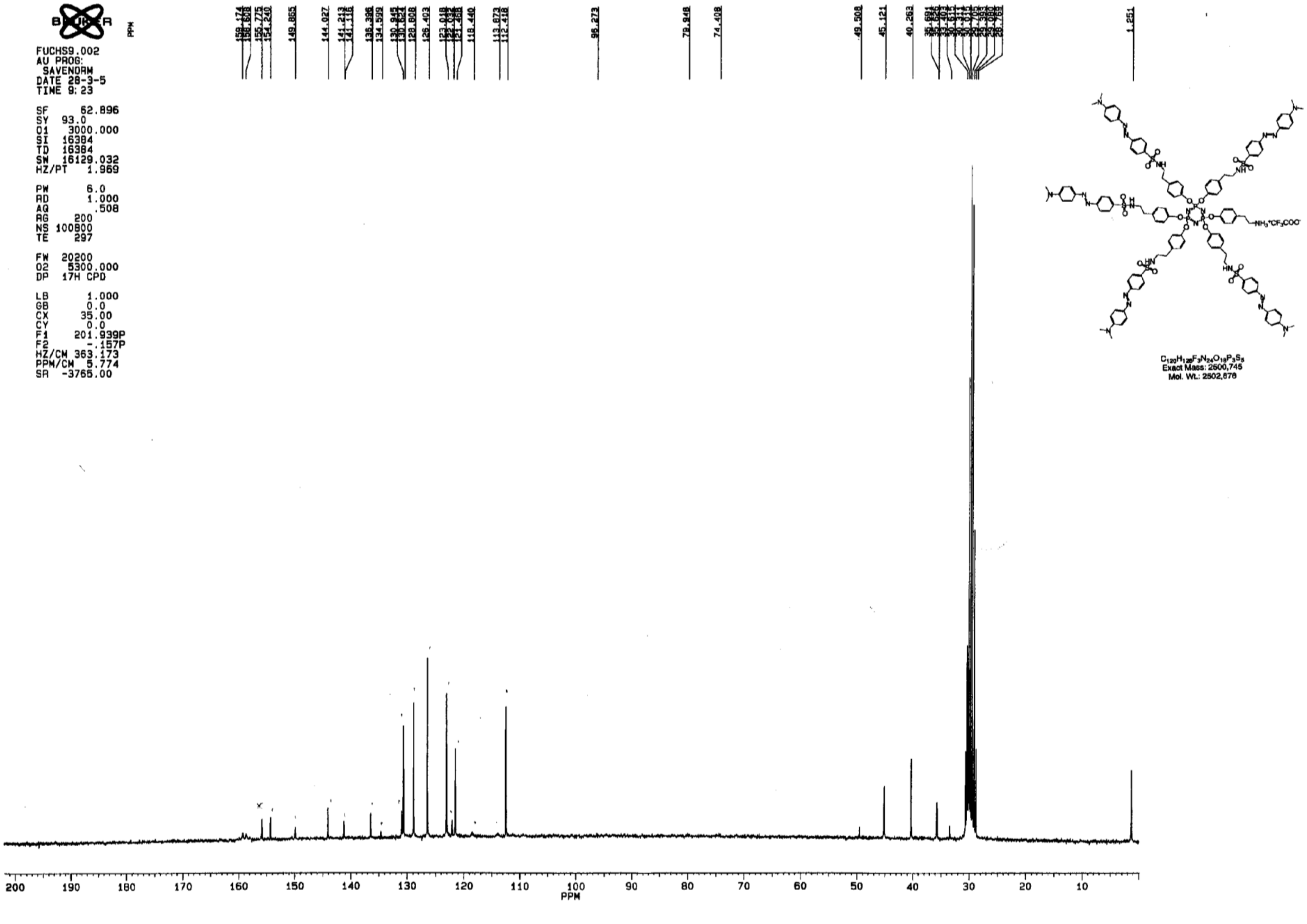

Mass spectrum (ion spray) of dendron **12**

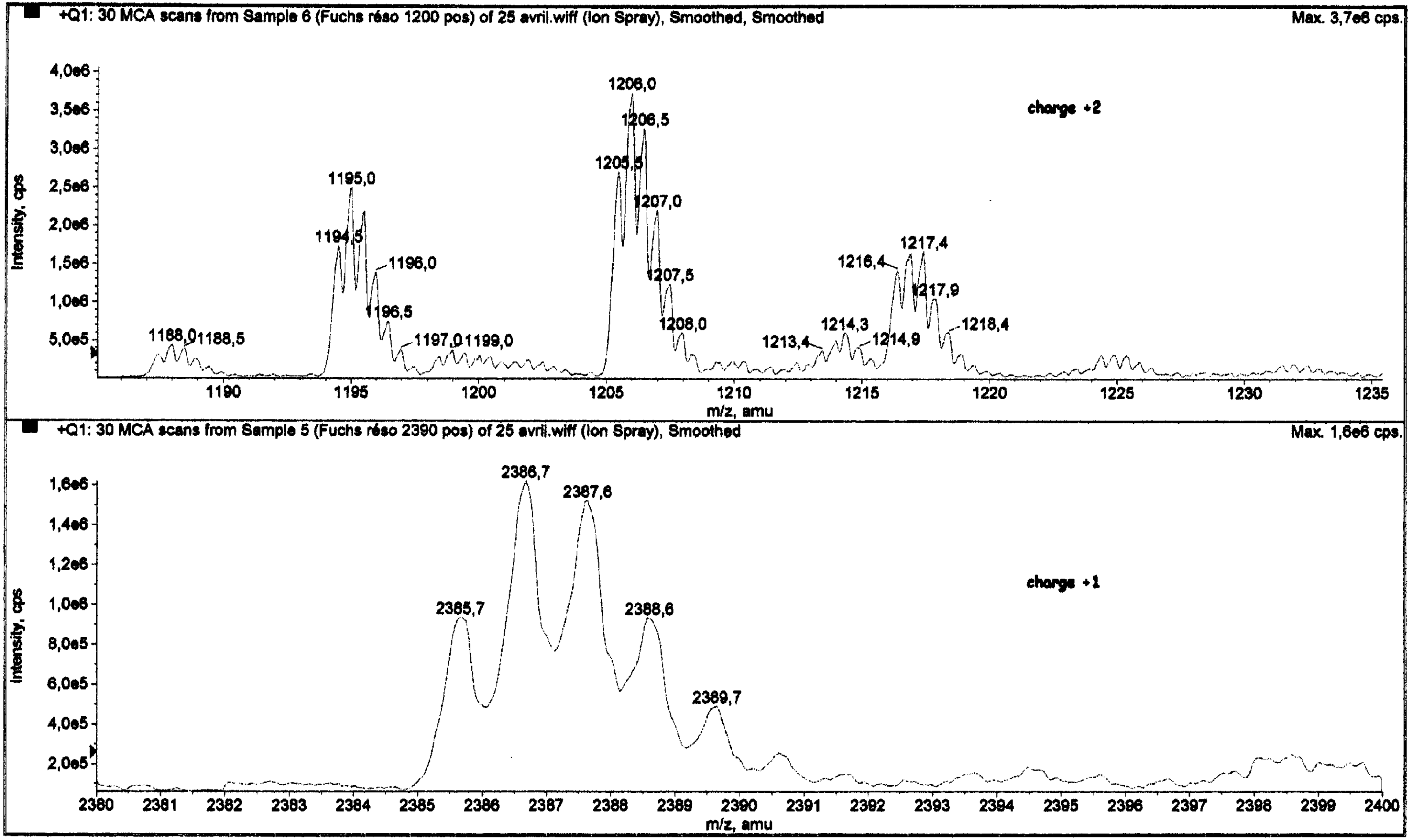

Supplement: File 2 — Spectral details. [file Beilstein_J_Org_Chem-07-1577-s002.pdf]
